# Telomere-to-telomere genome assembly of yellow-fruited allotetraploid American ginseng (*Panax quinquefolius* L*.*) provides insights into flavonoid biosynthesis

**DOI:** 10.1093/hr/uhaf198

**Published:** 2025-07-29

**Authors:** Xiu-Juan Lei, Jing Zhao, Jun-Bo Rong, Meng-Yang Zhang, Wen-Hao Jia, Jie Zhang, Xi Chen, Hui Hu, Jia Wu, Yi-Jie Jiang, Li-Wen Feng, Yi-Fei Wang, Michael K Deyholos, Li-Yao Su, Hui Liu, Peng Di, Jian Zhang, Ai-Sheng Xiong, Ying-Ping Wang

**Affiliations:** College of Chinese Medicinal Materials, Jilin Agricultural University, Changchun 130118, China; State Local Joint Engineering Research Centre of Ginseng Breeding and Application, Jilin Agricultural University, Changchun 130118, China; College of Chinese Medicinal Materials, Jilin Agricultural University, Changchun 130118, China; State Local Joint Engineering Research Centre of Ginseng Breeding and Application, Jilin Agricultural University, Changchun 130118, China; College of Chinese Medicinal Materials, Jilin Agricultural University, Changchun 130118, China; State Local Joint Engineering Research Centre of Ginseng Breeding and Application, Jilin Agricultural University, Changchun 130118, China; College of Chinese Medicinal Materials, Jilin Agricultural University, Changchun 130118, China; State Local Joint Engineering Research Centre of Ginseng Breeding and Application, Jilin Agricultural University, Changchun 130118, China; College of Chinese Medicinal Materials, Jilin Agricultural University, Changchun 130118, China; State Local Joint Engineering Research Centre of Ginseng Breeding and Application, Jilin Agricultural University, Changchun 130118, China; College of Chinese Medicinal Materials, Jilin Agricultural University, Changchun 130118, China; State Local Joint Engineering Research Centre of Ginseng Breeding and Application, Jilin Agricultural University, Changchun 130118, China; College of Chinese Medicinal Materials, Jilin Agricultural University, Changchun 130118, China; State Local Joint Engineering Research Centre of Ginseng Breeding and Application, Jilin Agricultural University, Changchun 130118, China; College of Chinese Medicinal Materials, Jilin Agricultural University, Changchun 130118, China; State Local Joint Engineering Research Centre of Ginseng Breeding and Application, Jilin Agricultural University, Changchun 130118, China; College of Chinese Medicinal Materials, Jilin Agricultural University, Changchun 130118, China; State Local Joint Engineering Research Centre of Ginseng Breeding and Application, Jilin Agricultural University, Changchun 130118, China; College of Chinese Medicinal Materials, Jilin Agricultural University, Changchun 130118, China; State Local Joint Engineering Research Centre of Ginseng Breeding and Application, Jilin Agricultural University, Changchun 130118, China; College of Chinese Medicinal Materials, Jilin Agricultural University, Changchun 130118, China; State Local Joint Engineering Research Centre of Ginseng Breeding and Application, Jilin Agricultural University, Changchun 130118, China; Faculty of Agronomy, Jilin Agricultural University, Changchun 130118, China; Department of Biology, University of British Columbia, Okanagan, Kelowna, BC V1V 1V7, Canada; College of Horticulture, Nanjing Agricultural University, Nanjing 210095, China; College of Horticulture, Nanjing Agricultural University, Nanjing 210095, China; College of Chinese Medicinal Materials, Jilin Agricultural University, Changchun 130118, China; State Local Joint Engineering Research Centre of Ginseng Breeding and Application, Jilin Agricultural University, Changchun 130118, China; College of Chinese Medicinal Materials, Jilin Agricultural University, Changchun 130118, China; Faculty of Agronomy, Jilin Agricultural University, Changchun 130118, China; Department of Biology, University of British Columbia, Okanagan, Kelowna, BC V1V 1V7, Canada; College of Horticulture, Nanjing Agricultural University, Nanjing 210095, China; College of Chinese Medicinal Materials, Jilin Agricultural University, Changchun 130118, China; State Local Joint Engineering Research Centre of Ginseng Breeding and Application, Jilin Agricultural University, Changchun 130118, China

## Abstract

*Panax quinquefolius* L., commonly known as American ginseng, is a valuable beneficial medicinal herb renowned for its health-promoting properties and rich phytochemical profile. Despite significant progress in understanding ginsenoside biosynthesis, the genetic basis for flavonoid diversity in American ginseng remains unclear. This study reports the first telomere-to-telomere (T2T) genome assembly for yellow-fruited American ginseng cultivar ‘Zhongnongyangshen No. 2’. The genome assembly, achieved using PacBio HiFi and Oxford Nanopore Technology ultra-long read technologies, offers a high-quality reference for genomic research, addressing previous gaps in structural accuracy. Combining transcriptomic and metabolomic analyses, we investigated flavonoid biosynthesis and the regulatory mechanisms underlying fruit color variation during different developmental stages of American ginseng. Our findings highlight the phylogenetic evolution of the American ginseng genome and offer new insights into the biosynthetic pathways of anthocyanins and flavonols. This comprehensive genomic resource facilitates deeper exploration of flavonoid diversity, supports genetic improvement efforts, and enhances the potential for future applications in medicinal plant research.

## Introduction

*Panax quinquefolius* L., well known as American ginseng, is a perennial herbaceous plant in the Araliaceae family, native to North America. It was introduced to China in the early 18th century by French missionaries. Over the past 300 years, *P. quinquefolius* has been highly regarded in traditional Chinese medicine for its various health benefits, including strengthening the lungs, reducing internal heat, generating bodily fluids, and relieving fatigue [1]. While ginsenosides have long been recognized as the primary active components in ginseng species [2], recent studies have highlighted the significant health benefits of flavonoids—such as flavones, flavonols, anthocyanins, and other flavonoid compounds—in *P. quinquefolius*. These compounds are also appreciated in East Asia for their anti-inflammatory, analgesic, antioxidant, and cardiovascular benefits [3].

Flavonoids, as critical plant secondary metabolites, have a crucial role in growth, development, and self-defenses [4]. Flavonoids and flavonols are directly involved in processes like cell division, differentiation, and elongation, while anthocyanins contribute to pigmentation, enriching flowers, fruits, and other plant parts with vibrant colors [5]. This diverse array of flavonoids not only enhances the medicinal value of ginseng species but also contributes to their ornamental appeal, highlighting their significance for further research.

In recent years, a combination of multi-omics and biochemical analyses has enabled the identification of genes related to ginsenoside biosynthesis in *P. quinquefolius* [6]. However, the genetic basis for the rich flavonoid content in *P. quinquefolius* remains unclear. High-quality genome assembly is essential for genomic, genetic, and molecular function analyses. In 2022, first *P. quinquefolius* reference genome (v1.0) was sequenced and assembled, revealing its genome size of 3.6 Gb and a scaffold N50 of 138.6 Mb [7]. In 2024, a new chromosome-level genome assembly of *P. quinquefolius* was reported, expanding the genome size to 4.51 Gb with an improved scaffold N50 of 187.93 Mb, and anchoring all sequences to 24 pseudochromosomes [8]. While these assemblies of *P. quinquefolius* provide valuable insights and facilitated gene discovery, gaps still remain that limit in-depth exploration of complex genomic regions. With advances in sequencing technology, particularly PacBio HiFi [9] and Oxford Nanopore Technology (ONT) ultra-long reads [10], gap-free telomere-to-telomere (T2T) genome assemblies have been successfully achieved in other plant species such as ginseng [11] and grape [12]. These T2T assemblies not only close missing sequences and correct structural errors, but also enable precise analysis of inter-varietal and interspecies variations, thereby improving chromosomal mapping accuracy. In nature, the majority cultivated *P. quinquefolius* populations are admixed, which has posed significant challenges to the assembly of high-quality genomes. ‘Zhongnongyangshen No. 2’(ZN) *P. quinquefolius*, derived from four successive generations of strict selfing and selection, is currently the only homozygous variety of *P. quinquefolius*. Its typical stably inherited phenotypic characteristics across multiple generations are yellow fruits and entirely green plant parts. This homozygous trait makes ZN the ideal material for *P. quinquefolius* T2T genome sequencing and research on the biosynthetic pathways of secondary metabolite.

In present study, we assembled a T2T reference genome for the homozygous yellow-fruited *P. quinquefolius* cultivar ZN, using this enhanced, high-quality reference genome, we investigated the phylogenetic evolution of *P. quinquefolius* genome. Additionally, we explored the genetic basis for differences in anthocyanin and flavonol content across different fruit colors and developmental stages of *P. quinquefolius* by combining transcriptomic and metabolomic data. Analysis of the T2T reference genome and multi-omics data not only provides valuable resources for a comprehensive understanding of *P. quinquefolius* genome, but also lays a solid foundation for elucidating the biosynthetic pathways of flavonoid compounds and the genetic regulatory mechanisms underlying fruit color formation in *P. quinquefolius*. Presented study report a successful assembly of a gap-free, T2T genome for *P. quinquefolius*, achieved through the integration of data from multiple sequencing platforms. This prime genome assembly would definitely serve as a critical resource for advancing genetic and genomic research on this important medicinal plant.

## Results

### A T2T genome assembly of *P. quinquefolius*

**Figure 1:**
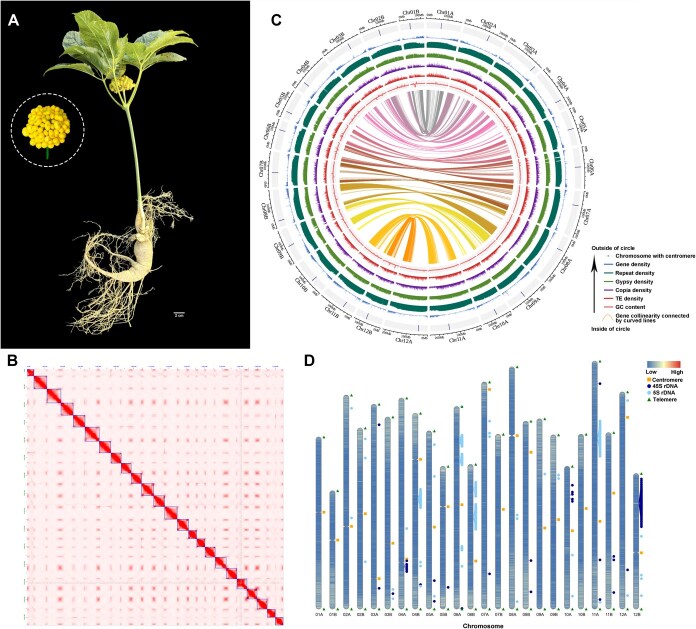
T2T gap-free genome assembly and genomic features of *P. quinquefolius*. **A** Morphological depiction of the *P. quinquefolius* plant and its yellow fruit. **B** Hi-C-based interaction heatmap of chromosomal fragments. **C** Genomic landscape of the *P. quinquefolius* assembly. The concentric circles (from outer to inner) illustrate: pseudochromosomes with centromeres (Chr01A–Chr12A and Chr01B–Chr12B), gene density, repeat density, Gypsy density, Copia density, TE density, GC content, and homologous gene collinearity within the genome. The chromosomes are depicted within a 50 kb window. **D** Gene density and the distribution of centromeres and telomeres are depicted in the T2T gap–free genome. Blue represents low, red represents high, the orange square marks the location of the centromere, dark blue indicates the position of 45S rDNA, aqua blue indicates the position of 5S rDNA, and blue triangles mark the locations of telomeres.

*P. quinquefolius* is indigenous to North America with a natural distribution across the United States and Canada. It was introduced to Jilin Province, China, in 1975. Subsequent cultivation, domestication, and selective breeding efforts have led to the development of a yellow-fruited variant, expanding the genetic diversity of this medicinally valuable species (Fig. 1A). Genome survey revealed that heterozygosity rate of *P. quinquefolius* was 1.18% (Table S1; Fig. S1). The T2T reference genome of *P. quinquefolius* (vT2T) was assembled with an integrated sequencing strategy that combined PacBio HiFi, ONT ultra-long reads, Illumina short reads, and Hi-C chromatin-conformation data. The PacBio HiFi dataset contributed 205.72 Gb of highly accurate reads (mean length = 17 741 bp; N50 = 17 719 bp), while the ONT ultra-long reads (read N50 = 97 kb) enhanced the resolution of complex repetitive regions (Table S2). Three initial assemblies were generated: (i) an HiFi-only assembly produced with hifiasm, (ii) an ONT-only assembly generated with NextDenovo, and (iii) HiFi + ONT assembly constructed with hifiasm (Table S3). The hybrid assembly exhibited the greatest contiguity, yielding a contig N50 of 185.33 Mb and a BUSCO completeness of 98.0%, outperforming the HiFi (contig N50 = 170.21 Mb; BUSCO = 97.9%) and ONT(contig N50 = 20.20 Mb; BUSCO = 94.7%) assemblies (Table S3).

Following short-read polishing with Illumina data, the consensus quality value (QV) of the updated *P. quinquefolius* vT2T assembly increased to 47.3. Hi-C scaffolding then anchored 4.33 Gb of sequence into 24 chromosome level pseudomolecules (Fig. 1B), yielding a scaffold N50 of 185.37 Mb, 33.8% higher than the v1.0 assembly (138.6 Mb, Table 1). All 48 telomeres and 24 centromeres were fully resolved, producing a truly gap-free T2T assembly (Fig. 1C). Compared with v1.0 assembly, the vT2T assembly shows substantial improvements across key metrics: genome size expanded from 3.6 Gb to 4.33 Gb, GC content rose from 34.11 to 35.91%, the contig count plummeted from 6762 to 24, and repeat content increased from 83.83 to 88.94%, including 83.21% long terminal repeats (Gypsy 57.03%, Copia 9.19%) (Table 1). BUSCO completeness reached 98.0% at the genome level and 99.4% for predicted proteins, outperforming the 93.93% protein BUSCO score of v1.0 assembly. The LTR Assembly Index (LAI) likewise climbed from 7.99 to 12.58 (Table 1). This chromosome-level, gap-free reference genome therefore provides a robust platform for functional and comparative genomics aimed at elucidating the biosynthetic pathways of bioactive compounds in *P. quinquefolius.*

**Table 1:** *P. quinquefolius* genome assembly statistics.

**Genome information**	***P.** quinquefolius* **vT2T**	***P.** quinquefolius* **v1.0**
Genome size (Gb)	4.33	3.60
GC content (%)	35.91	34.11
Number of contigs	24	6762
Scaffold N50 (Mb)	185.37	138.6
Number of protein-coding genes	67 641	64 247
Repeat sequence content (%)	88.94	83.83
LTR content (%)	83.21	80.68
Gypsy content (%)	57.03	52.20
Copia content (%)	9.19	7.27
Number of telomeres	48	\
Number of centromeres	24	\
QV	47.3	\
Complete genome BUSCO (%)	98.0	\
Complete protein prediction BUSCO (%)	99.4	93.93
LAI	12.58	7.99

### Telomeres and centromeres identifications

To assess the completeness of telomeres and centromeres within the *P. quinquefolius* genome, quarTeT was employed for telomere prediction and T2Tools for centromere prediction (https://github.com/sc-zhang/T2Tools). Predictive analyses of the genome sequence identified a total of 48 telomeres and 24 centromeres. (Fig. 1C and D; Table 1; Tables S4 and S5). The number of motifs within these repetitive sequences’ ranges from 1508 to 2796 (Table S4). The telomere lengths at the upstream ends of the chromosomes showed a variation from a minimum of 12.8 kb to a maximum of 19.9 kb, while those at the downstream ends range from 9.3 to 19.9 kb (Table S4). To identify potential centromeric regions on the chromosomes of *P. quinquefolius,* short tandem repeats were employed. Hi-C interaction heatmaps were integrated with large unannotated regions (Fig. 1B), areas of low gene density, and regions of high tandem repeats (TRs) density (Fig. 1C; Fig. S2), potential centromeres on 24 chromosomes were then successfully identified (Fig. 1D). The lengths of these potential centromeres fall within a range of 17.5 to 2766.9 kb (Table S5). It was also found that the potential centromere of Chr07A is located near the end of the chromosome.

### A T2T genome annotation of *P. quinquefolius*

The genome of *P. quinquefolius* has been annotated using a combination of *de novo* prediction methods, homology-based searches, and RNA-seq data. Gene annotation identified 67 741 protein-coding genes, a 5.2% increase compared to v1.0 assembly (64 247 genes), supported by a 98.67% RNA-seq read mapping rate (Table 1). The gene density distribution across chromosomes was observed to be uneven, with higher concentrations at the chromosomal ends (Figs 1C and S2).

Annotation of transposable elements (TEs) in the T2T genome revealed that repetitive sequences account for 88.94% of the *P. quinquefolius* genome, among which LTR elements are the most abundant, comprising 83.21% of the genome. Specifically, LTR/Gypsy and LTR/Copia constituted 57.03 and 9.19% of the T2T genome, respectively (Table 1). The spatial distribution of repetitive elements on chromosomes was found to be non-uniform, with lower concentrations towards the chromosomal ends (Figs 1C and S2).

In consideration of the uncertain or possibly extinct status of the diploid ancestors of *P. quinquefolius*, the species has been identified as an allotetraploid based on chloroplast and nuclear genes analysis [13]. We conducted a scan of all 19 bp sequences (19-mers) across the chromosomes and identified chromosome-specific 19-mers (Fig. S3A). The contigs resulting from the T2T assembly were organized into 24 pseudochromosomes, and two subgenomes (designated as A and B) were differentiated through the use of a combination of homologous genes and subgenome-specific k-mers. To mitigate potential biases introduced by extensive structural variations (SVs) during sequence similarity-based clustering, our analytical framework selectively interrogated syntenic chromosomal blocks. Consequently, two distinct groups, labeled as SG1 and SG2, were identified (Figs S3B–3D). Subgenomes A and B exhibited distinct assembly sizes of 2.38 and 1.95 Gb, respectively, with 34 110 and 33 631 genes in each (Table S6). Alignment of subgenome-specific k-mer datasets against LTR retrotransposons annotated by SubPhaser revealed differential association patterns: 21% (38 866/185018) of A-subgenome-specific k-mers corresponded to long terminal repeat retrotransposons (LTR-RTs), compared to 22.6% (41 121/181932) in the B-subgenome. Furthermore, subgenome-resolved LTR-RT annotation demonstrated proportional specificity, with 23.5% (40 069/170444) and 25.3% (43 139/170444) of elements being uniquely assigned to the A- and B-subgenomes, respectively. Divergence timing estimates for subgenome-resolved LTR-RT insertions demonstrated significant temporal heterogeneity, spanning 0.065 to 6.76 million years within the 95% confidence interval (Fig. S3E). The insertion times of subgenome-specific LTR-RTs were estimated, revealing earlier insertions in subgenome B compared to subgenome A (Fig. S3E). Phylogenetic analysis of LTR-RTs demonstrated that subgenome-specific elements diverged into phylogenetically distinct clades (Fig. S3F). Therefore, our k-mer-based phasing results are in agreement with the assembled A and B subgenomes and support the conclusion that *P. quinquefolius* is an allotetraploid.

### SVs and presence–absence variations in T2T gap-free genome

Compared to *P. quinquefolius* v1.0, the newly assembled T2T gap-free genome revealed a diverse range of SVs, including inversions, translocations, and duplications across all contigs, highlighting improved genome assembly quality (Fig. S4A). Among these SVs, duplication events made up the largest portion, representing 95% (65 845/91957) of the total (Fig. S4B). These duplications were primarily located in telomeric regions, while deletion and insertion variations accounted for 14.9% (13 189/91957) and 12.7% (9540/91957) of the SVs, respectively.

Gene Ontology and KEGG analyses were applied to 5909 genes located in SV regions and 1508 genes of presence variation regions and 1665 genes of absence variation regions (Table S7). SV genes are preferentially enriched for sequence—specific DNA-binding functions that regulate RNA polymerase II initiation, together with DNA recombination, nucleotide—excision repair, the mRNA surveillance pathway and aminoacyl tRNA biosynthesis pointing to a role in post-transcriptional quality control and protein synthesis. presence–absence variations (PAVs) genes share the DNA-binding signal but are dominated by basal transcription factor (TF) activity and microtubule-based movement, and map most strongly to the KEGG basal TF module alongside multiple metabolic and repair pathways. Collectively, genes of SVs mainly influence downstream processing, whereas genes of PAVs target the core transcriptional machinery and broader metabolic networks (Fig. S4C–F).

### Phylogenomic and evolution of *P. quinquefolius*

**Figure 2:**
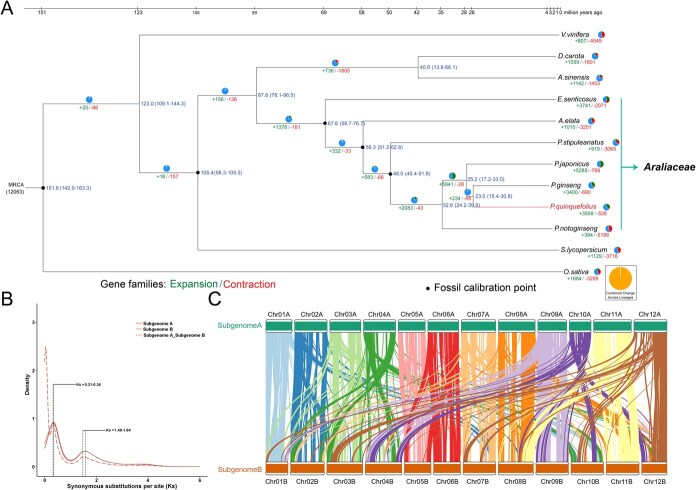
Comparative genomic insights between *P. quinquefolius* and other species. **A** Evolutionary Insights: Phylogeny, Divergence, and Gene Family Changes in 12 Species. Divergence times are presented in millions of years ago (Mya) and are annotated next to each node on the phylogenetic tree. Contractions and expansions of gene families are represented by red and green, respectively. **B** Plot of Synonymous Substitutions per synonymous site (ks) for detecting WGD events in *P. quinquefolius*. **C** Colinear genome comparison between A- and B-subgenomes of *P. quinquefolius* at the gene level.

To elucidate evolutionary characteristics and gene families of *P. quinquefolius*, the T2T genome was compared with those of eleven previously sequenced plant genomes, utilizing the angiosperm *Oryza sativa* as the outgroup for phylogenetic analysis. Employing a robust clustering approach for gene family analysis, we identified a total of 69 321 gene families. Notably, 7973 of these gene families were identified as ubiquitous, occurring in all species examined, and collectively encompassed 214 285 genes. Specifically, *P. quinquefolius* harbored 4377 unique gene families, comprising 5053 genes (Fig. S5; Table S8). A comparative assessment was undertaken involving *P. quinquefolius* and three additional species belonging to the Araliaceae family (*Panax. ginseng*, *Panax. japonicus*, and *Panax. notoginseng*). The four *Panax* species shared 13 643 gene families, with *P. quinquefolius* possessing 6292 exclusive gene families (Fig. S6A). GO enrichment analysis revealed that the unique gene families in *P. quinquefolius* were associated with terpenoid biosynthetic process (Fig. S6B). Compared to the most recent common ancestor (MRCA) of the twelve plant species, 3508 gene families (15 004 genes) in *P. quinquefolius* gone expansion, while 528 gene families (1062 genes) shown contraction (Fig. 2A). KEGG pathway enrichment analysis demonstrated that the distinctive gene families present in *P. quinquefolius* were linked to the biosynthesis of flavonoids (Fig. S7). Phylogenetic tree analysis was constructed based on a superalignment matrix derived from 39 single-copy orthologs across 12 species. Notably, *Eleutherococcus senticosus* (Eleutherococcus) diverged from Aralia approximately 67.6 million years ago (Mya), whereas *Aralia elata* (Aralia) separated from *Panax* around 56.3 Mya. Subsequently, *P. stipuleanatus*, *P. notoginseng*, and *P. japonicus* branched off from *Panax* at approximately 48.0, 32.6, and 25.2 Mya, respectively. These findings support previously proposed phylogenetic sequences (Fig. 2A).

As *P. quinquefolius* is an allotetraploid plant, a 19-Kmer-based clustering method was employed to divide its chromosomes into two subgenomes (Fig. S3), which exhibited good covariance (Fig. 1B). Subgenome A was found to have 3411 expanded gene families and 281 contracted gene families, whereas Subgenome B possessed 3423 expanded gene families and 321 contracted gene families (Table S9). However, it was noted that 3326 expanded gene families were commonly present in both subgenomes A and B, and 184 contracted gene families were shared between the two subgenomes (Fig. S8; Table S9).

To explore the occurrence of whole-genome duplication (WGD) events in the evolutionary history of *P. quinquefolius*, we performed an analysis of the distribution of synonymous substitution rates (Ks values) among homologous genes, comparing *P. quinquefolius* with two other species within the *Panax* genus. (Fig. S9A). The outcomes demonstrated the presence of two prominent peaks in the vicinity of 0.2–0.3 and 1.46–1.47 in pairwise comparisons among any two of these species (Fig. S9A). This observation indicates that all three *Panax* species studied have undergone these two genome-wide duplication events, thereby marking significant milestones in the evolutionary trajectory of *P. quinquefolius*. To delve into the WGD events in the evolutionary trajectory of *P. quinquefolius*, we analyzed the distribution of Ks values among homologous genes, specifically comparing the A and B subgenomes within this species. Both subgenome A and subgenome B displayed two prominent peaks in the range of 0.31–0.34 and 1.49–1.64, respectively, suggesting that both subgenomes have undergone two common WGD events. The comparison of Ks values among homologous genes between subgenome A and B revealed a unique peak at approximately 0.02–0.03 that was not observed within either subgenome A or B individually (Fig. 2B). Collinearity analysis conducted on subgenome A and subgenome B revealed continuous collinearity among some homeologous chromosome pairs. Chr01A/B, Chr02A/B, Chr05A/B, Chr06A/B, Chr07A/B, Chr08A/B, Chr09A/B, and Chr12A/B show a high degree of collinearity. In contrast, Chr10A and Chr10B share little collinearity, a large inverted segment on Chr10B aligns with the distal region of Chr01A, suggesting that this region was first translocated from Chr01A to Chr10B and then inverted, thereby creating the pronounced structural asymmetry between the Chr10 homeologous chromosome pairs. Collinearity between Chr04A and Chr04B is similarly low, and the presence of inverted segments between Chr04A, Chr03B and Chr03A, Chr04B indicates asymmetric inter-chromosomal rearrangements. Chr11A and Chr11B remain largely collinear, with only minor local inversions, reflecting overall structural conservation. Finally, partial collinearity between Chr06A, Chr07B and Chr05A, Chr06B points to additional small-scale rearrangements (Fig. S9B).

### Analysis of flavonoid biosynthetic pathway of *P. quinquefolius*

**Figure 3:**
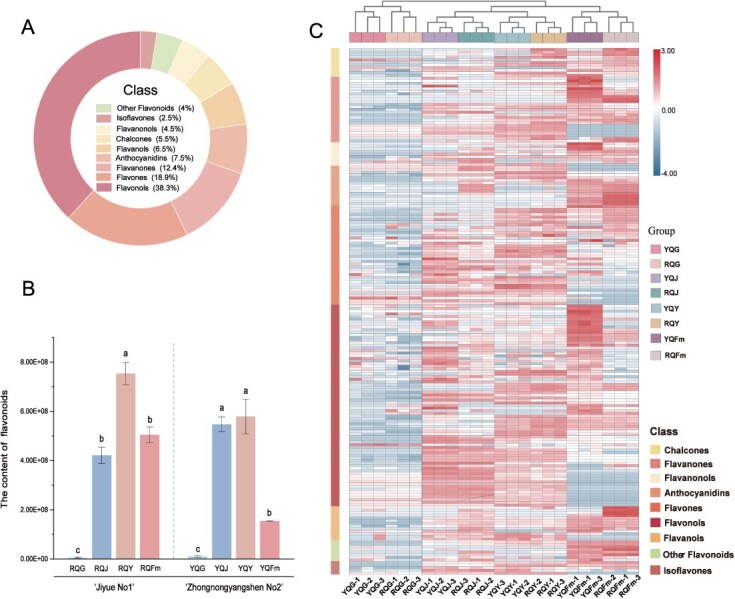
Flavonoid metabolites in different tissues of *P quinquefolius.*  **A** Circular diagram showing the metabolite composition of the flavonoids of *P. quinquefolius*. Each color represents a category, and the area of the colored block indicates its proportion. **B** Flavonoid content in roots, stems, leaves and fruits of *P. quinquefolius*. **C** Hierarchical clustering of flavonoid content of two *P. quinquefolius* varieties at different tissues. YQG (ZN roots), YQJ (ZN stems), YQY (ZN leaves), YQFm (ZN fruits), RQG (JY roots), RQJ (JY stems), RQY (JY leaves), and RQFm (JY fruits).

Flavonoids are a class of important bioactive compounds that possess a wide range of pharmacological activities and play an irreplaceable role in plant growth and development. Contents of flavonoid metabolites and the related genes expression were examined in four tissues (root, stem, leaf, and fruit) of two *P. quinquefolius* varieties (ZN, yellow-fruited; ‘Jiyue No. 1’(JY), red-fruited), and a total of 201 flavonoid metabolites were identified (Table S10). These metabolites could be divided into nine categories (Fig. 3A): flavonols, flavones, flavanones, anthocyanins, flavanols, chalcones, flavanonols, isoflavones, and other flavonoids, among which flavonols had the highest content and the largest variety in the two varieties (Fig. S10). In addition, one-way ANOVA (*P* < 0.05) and Tukey’s *post hoc* test were conducted for different tissue types (leaves, roots, stems, and fruits) in both varieties. The results showed that total flavonoid content was significantly highest in leaves and lowest in roots for both varieties (Fig. 3B). A cluster analysis of flavonoids identified in four tissues of the two varieties was conducted, and the results were shown in the heatmap (Fig. 3C). In the same tissue types, the flavonoid components in stems and fruits showed significant differences between the two varieties (Table S10) (stems: 92 differential metabolites; fruits: 115 differential metabolites), while the differences in roots and leaves were relatively smaller (roots: 45 differential metabolites; leaves: 56 differential metabolites). Further analysis of the top 20 different accumulated flavonoid in fruits (Fig. S11) showed that 13 components were found to have a high content in the fruits of ZN, including ten flavonols, two flavanones and one flavanonol. The components with higher content in the fruits of JY were three flavanols, two anthocyanins, one flavonol, and one chalcone.

**Figure 4:**
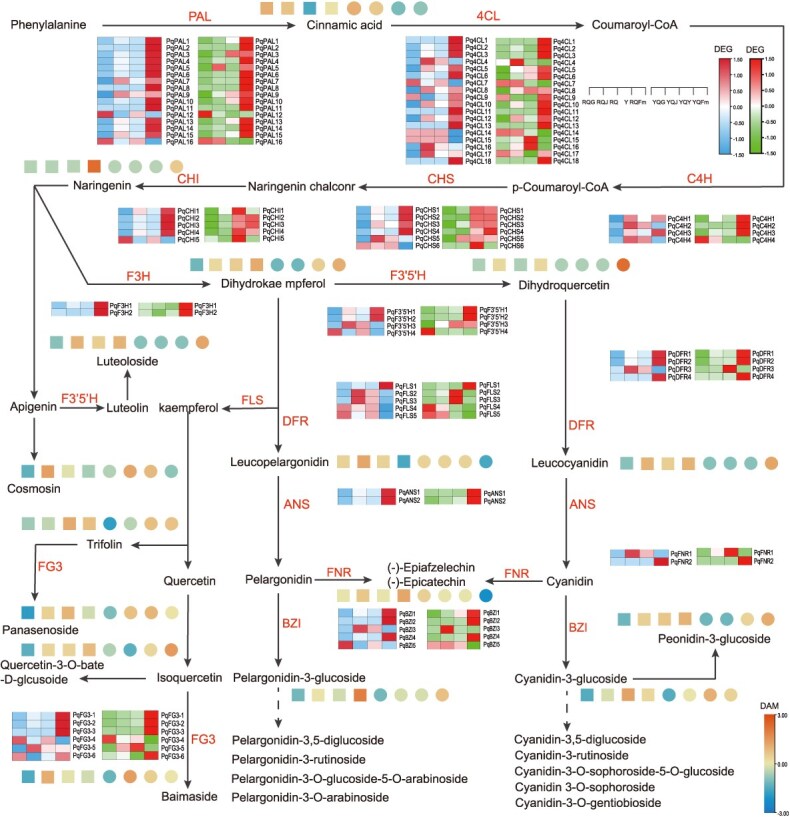
Genes involved in flavonoid biosynthesis. Heatmap showing gene expression levels and corresponding metabolite content in different tissues. The two heatmaps represent JY (the former) and ZN (the latter), and the horizontal coordinates in the heatmaps represent the four tissues of roots, stems, leaves and fruits from left to right, and the dot heatmaps indicate the content of the flavonoids in different tissues. The dot plot heatmap distinguishes the two varieties by marker shape: squares denote JY, and circles denote ZN. The color intensity corresponds to the level of flavonoids content.RQG (JY roots), RQJ (JY stems), RQY (JY leaves), RQFm (JY fruits), YQG (ZN roots), YQJ (ZN stems), YQY (ZN leaves), and YQFm (ZN fruits).

By using this *P. quinquefolius* T2T genome, 140 candidate genes related to flavonoid biosynthesis were identified (Table S11), which may be involved in multiple enzymatic reaction steps in the flavonoid biosynthesis pathway (Fig. 4; Table S12). The biosynthesis of flavonoids is initiated from the phenylpropanoid pathway. Our findings indicate that most phenylpropanoid biosynthesis genes (*PqPALs*, *Pq4CLs*, *PqC4Hs*) are predominantly expressed in the fruits. However, certain genes, including *PqPAL16*, *Pq4CL15*, *Pq4CL16*, *Pq4CL17*, *PqC4H2,* and *PqC4H4* display distinct tissue-specific expression patterns between the two varieties (Fig. 4). Naringenin serves as a pivotal precursor in the biosynthesis of flavonoids, participating in the subsequent diversification of these compounds. Our investigation revealed a significant disparity in naringenin content between the fruits of ZN and JY, with the JY (red fruit) containing 10.24 times more naringenin than the ZN (yellow fruit). Transcriptomic analysis indicated that in JY, the genes *PqCHI1*, *PqCHI2*, *PqCHI3*, and *PqCHI4* were highly expressed in the fruit, whereas in the ZN, *PqCHI1*, and *PqCHI4* exhibited higher expression levels in the leaves. These differences in gene expression likely underlie the differential accumulation of naringenin in the fruits of the two varieties. Furthermore, *F3H*, *F3′5′H*, *FLS*, *DFR*, and *ANS* are key branch-point enzymes in flavonoid biosynthesis. The F3H, F3*′*5*′*H and FLS are involved in the synthesis of dihydroflavonols and flavonols (luteolin, dihydroquercetin, and their derivatives), while the DFR and ANS primarily contribute to the formation of anthocyanins. In this study, the expression patterns of *PqF3′5′H3*, *PqDFR3*, *PqFLS2*, and *PqFLS3* exhibited significant tissue-specific differences between ZN and JY. In ZN, these genes were predominantly highly expressed in the leaves, whereas in JY, their expression levels were not only maintained at high levels in the leaves but also significantly upregulated in the stems. These differences in gene expression may account for the distinct accumulation of flavonoids in the stems of the two varieties.

**Figure 5:**
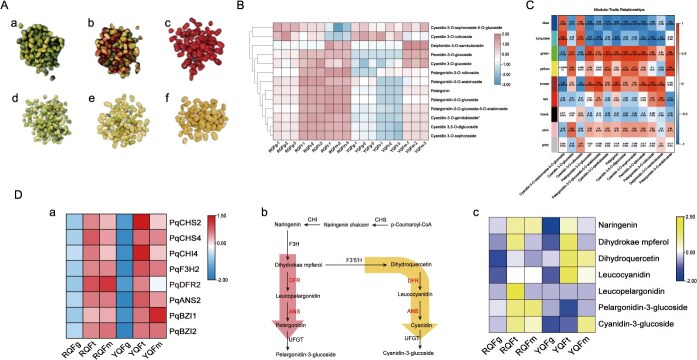
Anthocyanin content and related gene expression analysis during fruit development of two *P. quinquefolius* varieties. **A** Fruit phenotype in three periods of *P. quinquefolius*. a-c，RQFg (JY green fruiting stage), RQFt (JY turning stage), RQFm (JY ripening stage), and d-f, YQFg (ZN green fruiting stage), YQFt (ZN turning stage), and YQFm (ZN ripening stage). **B** Changes in the content of anthocyanin components during fruit development. **C** Weighted gene co-expression network analysis of gene expression and anthocyanin composition in fruits of JY at different developmental stages. **D** Expression of anthocyanin synthesizing genes and regulated metabolites in the blue module in two varieties of *P. quinquefolius*. **a** The heatmap illustrates the expression patterns of nine anthocyanin biosynthetic genes across different developmental stages of fruits in the two varieties. **b** The anthocyanin biosynthetic pathway is shown, with red arrows indicating the primary metabolic flux in JY fruits during the turning and ripening stages, and yellow arrows indicating the primary metabolic flux in ZN fruits during the turning and ripening stages. **c** The heatmap depicts the variations in the content of metabolites associated with anthocyanin biosynthesis across different developmental stages of fruits in the two varieties.

During the development of *P. quinquefolius* fruits (green fruiting stage, turning stage, and ripening stage) (Fig. 5A), through KEGG enrichment analysis using the hypergeometric test (*q*-value <0.05), we identified that a total of 22 differentially accumulated flavonoid metabolites were significantly enriched in the flavonoid biosynthesis pathway (Table S13). These metabolites mainly included flavonoid backbone precursors (naringenin, dihydrokaempferol, dihydroquercetin), anthocyanidin aglycones (pelargonidin-3-O-glucoside, cyanidin 3-O-glucoside), quercetin, and its glycoside derivatives (isorhamnetin, luteolin, vitexin). The anthocyanin accumulation shows significant differences between the two varieties.

Anthocyanin content and accumulation patterns differed significantly between the two varieties, with JY exhibiting substantially higher accumulation than ZN. In JY, anthocyanin levels increased sharply from the turning stage and remained high until ripening, whereas in ZN, accumulation began only at the ripening stage, resulting in significantly lower levels compared to JY. Dihydroquercetin and its glycosylated derivatives significantly accumulated during fruit development in ZN. For example, the content of isorhamnetin in ZN fruits was 4.88 times higher than that in JY fruits. Transcriptome analysis further identified 36 differentially expressed genes (DEGs) involved in the fruit development of *P. quinquefolius* (Table S14), correlation analysis between these DEGs and the differentially accumulated flavonoids revealed that the 22 flavonoid metabolites were divided into two clusters (Fig. S12). Naringenin, catechin, pelargonidin-3-O-glucoside, and cyanidin 3-O-glucoside were significantly positively correlated with *PqCHI1*, *PqCHI2*, *PqDFR1*, *PqDFR2*, and *PqANS1*. In contrast, dihydrokaempferol, dihydroquercetin, and their various glycosylated derivatives were significantly positively correlated with *PqCHS1, PqCHS2*, *PqCHS3*, *PqCHI3*, *PqCHI4*, *PqF3′5′H1* and *PqF3′5′H2* (Fig. S12).

The expression of genes at different fruit developmental stages related to flavonoid biosynthesis was analyzed in the fruits of two varieties. The results showed (Fig. S13) that during the development of JY fruits, the expression levels of the genes *PqPAL4*, *PqPAL5*, *PqPAL6*, *PqPAL15*, *Pq4CL5*, *Pq4CL12*, and *PqC4H1* increased in the turning stage and then decreased in the ripening stage. In contrast, in ZN fruits, the expression levels of *PqPAL4*, *PqPAL6*, *PqPAL15*, *Pq4CL5*, *Pq4CL12*, and *PqC4H1* continued to rise throughout the entire developmental period, while the expression level of *PqPAL5* declined progressively. Additionally, the genes *PqDFR1*, *PqDFR2*, and *PqANS1* exhibited a gradually increasing trend in the development of JY fruits, whereas in ZN fruits, their expression levels increased in the turning stage and then decreased in the ripening stage. The expression level of *PqF3′5′H2* increased during the turning stage and then decreased during the ripening stage in JY fruits, while in ZN fruits, the expression level of *PqF3′5′H2* continued to rise throughout the entire developmental period. The differential expression of *PqDFR1, PqDFR2, PqANS1*, and *PqF3′5′H2* likely underlies the varietal differences in flavonoid metabolite accumulation in the two varieties.

### Study on the mechanism of fruit coloration of *P. quinquefolius*

A total of 13 anthocyanins were detected in the two varieties, which could be divided into four classes according to their chemical structure, pelargonidin, cyanidin, peonidin, and delphinidin (Table S15). Comparative analysis of anthocyanin content across developmental stages (Fig. 5A) revealed significantly higher total anthocyanins in JY versus ZN fruits at all stages. In JY, pelargonidin and cyanidin accumulated rapidly during turning and stabilized at ripening, whereas in ZN, these compounds decreased during turning before increasing at ripening (Fig. 5B).

Weighted gene co-expression network analysis (WGCNA) was independently performed on DEGs datasets from the fruit developmental stages of JY and ZN varieties. In JY, nine significant co-expression modules were identified, with the green module showing the highest correlation with anthocyanin metabolites (Fig. 5C). KEGG enrichment analysis revealed that genes in this module were significantly enriched in pathways such as oxidative phosphorylation, the tricarboxylic acid cycle, anthocyanin biosynthesis, and flavonoid biosynthesis (Fig. S15A). Within this module, eight key anthocyanin synthesis genes were identified, including *PqCHS2*, *PqCHI4, PqF3H2*, *PqDFR2,* and *PqANS2* (Table S16).

In ZN fruits, the blue module exhibited the highest correlation with anthocyanin metabolites (Fig. S14). Nonetheless, its functions were primarily enriched in pathways related to unsaturated fatty acid synthesis, fatty acid metabolism, phenylpropanoid synthesis, and anthocyanin synthesis (Fig. S15B). Notably, key anthocyanin biosynthesis genes such as *CHI*, *DFR*, and *ANS* were absent from this module, consistent with the previous metabolomic data showing the absence of anthocyanins in ZN fruits. The expression patterns of these eight genes during fruit development in both varieties were further analyzed. It was found that the *PqDFR2* gene exhibited different expression patterns between the two varieties (Fig. 5Da). During the development of JY fruits, the expression level of this gene showed a continuous upward trend. In contrast, in ZN fruits, the expression levels of this gene increased in the turning stage but decreased in the ripening stage.

Dihydroflavonol-4-reductase (DFR) is a key enzyme in plant anthocyanin biosynthesis pathway, which can catalyze flavanonols substances to produce different anthocyanin precursors, and then catalyze anthocyanin precursors to produce anthocyanin under the action of anthocyanin synthetase (ANS) (Fig. 5Db). Therefore, DFR and ANS directly regulate anthocyanin composition and accumulation levels, thereby determining plant tissue coloration. Metabolic profiling of flavanonols, anthocyanin precursors, and anthocyanins across developmental stages revealed distinct accumulation patterns between varieties. JY fruits exhibited substantial accumulation of both anthocyanin precursors and anthocyanins (leucopelargonidin and pelargonidin-3-O-glucoside) during fruit development. In contrast, ZN fruits showed low levels of these compounds but significantly higher accumulation of dihydroquercetin (a flavanonol) compared to JY (Fig. 5Dc). Combined with transcriptome analysis, we speculated that the differences in the expression of *DFR* gene in the fruits of the two varieties may be the causes of fruit color differentiation between the two varieties.

## Discussion

With the rapid advancement of genome sequencing and assembly technologies, an increasing number of complex genomes, particularly among the medicinal plants, have been assembled and published. This holds significant importance for investigating the biosynthetic pathways of active compounds and genetic diversity within these species. *P. quinquefolius*, belonging to the Araliaceae, is a widely used traditional herbal medicine globally, possessing both biological and economic value. Having a highly continuous and complete reference genome in place is crucial for its genetic and genomic studies. However, the published genomes of *P. quinquefolius* are all at the chromosome level, yet to achieve the T2T level, which diminishes their value as reference genomes. Therefore, to enhance the quality of the *P. quinquefolius* genome and enrich its resources, this study conducted a *de novo* T2T genome assembly for the allotetraploid ZN—a homozygous yellow-fruited *P. quinquefolius*—and successfully disassembled it into two haplotype-resolved subgenomes at the reference genome level (Fig. S2). Leveraging the complementarity of different assembly strategies and splicing methods facilitated a more continuous genome assembly. The contig N50 (185.37 Mb) and BUSCO (99.4%) evaluation results of this genome are substantially higher than those of the *P. quinquefolius* v1.0 (contig N50: 138.6 Mb, BUSCO: 93.93%). Moreover, the completeness of this genome at the chromosome level far surpasses the published *P. quinquefolius* v1.0 and represents the most complete reference genome among the published *P. quinquefolius* genomes (Table 1). The complexity of heterochromatin and repetitive sequences makes centromeric and telomeric sequences the most challenging task in genome assembly. Through the integration of multi-omics data, we successfully predicted telomeric and centromeric sequences as well as candidate regions in the T2T *P. quinquefolius* genome, providing a genetic foundation for subsequent in-depth studies on centromeres and highly repetitive regions (Fig. S2). Additionally, we employed various strategies to annotate structural genes, repetitive sequences, non-coding RNAs, and gene functions in the T2T *P. quinquefolius* genome (Table 1; Figs S4–S7). The high-quality genome assembly of the yellow-fruited *P. quinquefolius* provides invaluable genetic resources for exploring the biosynthetic and metabolic pathways of flavonoids and the genetic regulatory mechanisms underlying fruit color formation.

Anthocyanins are synthesized via the flavonoid biosynthetic pathway. Variations of genes expression related to anthocyanin synthesis typically result in differences in anthocyanin accumulation, which is a major determinant of the diverse colors observed in plants [14, 15]. Many studies have shown that the formation of fruit color is closely related to the accumulation of anthocyanins. Ruiyi et al. [16] found that the accumulation of anthocyanins in dragon fruit determines the color changes of peel and flesh. Our study confirmed that the difference of anthocyanin content in the two *P. quinquefolius* varieties lead the difference in fruit color, which mainly appeared in the turning stage and the ripening stage. Among the genes related to anthocyanin synthesis, *DFR* and *ANS* showed different expression trends in the two varieties. *Arabidopsis thaliana* and tobacco have confirmed that overexpression of *DFR* gene can increase the content of anthocyanins in plants, thereby affecting color changes [17]. During the maturation of Chinese bayberry, rapid color changes occur, making it an ideal fruit for studying fruit coloration. The study found that the total anthocyanin content in purple fruit was 8.51 times that of white fruit, and the expression levels of *DFR1*, *DFR2*, *UFGT*, and regulatory factor *MYB1* gradually increased during the development stage of purple fruit, which was significantly higher than that of white fruit. Moreover, the expression of *MYB1* and *bHLH1* regulators in white fruits was not up-regulated [18]. In this study, we also found that three MYB genes and two bHLH genes were positively correlated with gene expression levels of *DFR* and *ANS* (Fig. s16; Table S17). Homologous comparison results showed that the three MYBs were homologous genes of *A. thaliana AtMYB75*, *AtMYB7,* and *AtMYB5*. These three genes can form complexes with bHLH TFs and co-regulate the expression of genes related to anthocyanin synthesis [19]. However, the expression trend of related TFs in *P. quinquefolius* was the same during the fruit development of two *P. quinquefolius* varieties, which may not be the main reason for the difference in fruit color between the two varieties. In order to further explore the relationship between *DFR* gene and fruit color change, we used the *P. quinquefolius* T2T genome and the *P. quinquefolius* v1.0 genome [7] to locate the *DFR* genes, respectively. The two *DFR* genes were precisely mapped to chromosomes 6 and 22 in the T2T genome assembly, exhibiting high sequence similarity. Notably, only one *DFR* locus was identified in the previous v1.0 genome assembly, demonstrating the superior resolution of the T2T approach. Specifically, the nucleotide sequence of the *DFR* gene extracted from the T2T genome differs from that in the v1.0 genome. This discrepancy may account for the differences in *DFR* gene expression patterns observed between the two *P. quinquefolius* varieties. The eggplant fruit color mutant (efc1) was artificially induced through EMS mutagenesis and is characterized by the near-absence of anthocyanins, resulting in white flowers and green fruits. Very low anthocyanin content was detected in various tissues of this mutant. Research has demonstrated that this phenotype is caused by a mutation in the *DFR* gene. The fruit color of the efc1 mutant can be restored through transgenic overexpression of the wild-type *DFR* transcript [20]. In *P. quinquefolius*, a similar mechanism may exist. The yellow-fruited variety of *P. quinquefolius* is the result of natural mutations during plant evolution. Besides exhibiting yellow fruits, its stems are stably green. In contrast, the red-fruited *P. quinquefolius* exhibits stem color polymorphism, displaying not only green but also purple and intermediate semi-purple pigmentation. Through transcriptome analysis of different tissue parts of the two *P. quinquefolius* varieties (Fig. S17), we found that the expression level of *DFR* gene in the fruit and stem of JY was significantly higher than that in ZN, but there was little difference in the expression level of root. The change of expression difference may be the main factor affecting the change of fruit color of *P. quinquefolius*.

## Materials and methods

### Plant materials

In 2024, 4-year-old red-fruited American ginseng (JY) and yellow-fruited American ginseng (ZN) from Fusong Ginseng Germplasm Resource Park, Jilin, China. ZN was used for genomic sequencing. The different tissue parts of JY and ZN (roots, stems, leaves, and fruits) were separated, where the fruits were sampled at three stages: green fruiting stage (with green fruit color), turning stage (with fruit color changing from green to red or yellow), and ripening stage (with red or yellow fruit color), respectively. The samples were prepared and subjected to metabolome assay and transcriptome sequencing on ultra-performance liquid chromatography–tandem mass spectrometry (UPLC-MS/MS) and Illumina platforms at Metware Biotechnology (Wuhan, China). Metabolome assay and transcriptome sequencing were performed on UPLC-MS/MS and Illumina platforms at Metware Biotechnology (Wuhan, China).

### Genome sequencing and assembly

ONT ultra-long raw sequencing reads were initially screened to eliminate those with an average quality score below 7. Subsequently, further filtering was applied using Filtlong (v0.2.4) to exclude fragments shorter than 10 kb. Adapter sequences were subsequently removed using Porechop (v0.2.4). Further filtering with Filtlong retained reads that were ≥ 30 kb in length and possessed mean read quality scores exceeding 90%. These high-quality reads were then utilized for the assembly process. For PacBio HiFi data, the circular consensus sequencing (CCS) software (v6.0.0) was applied to filter out subreads with fewer than three passes and a signal-to-noise ratio (SNR) below 2.5, yielding CCS reads for subsequent analysis. Second-generation sequencing data underwent preprocessing with fastp (v0.21.0) [fastp: an ultra-fast, comprehensive FASTQ preprocessor] to exclude low-quality reads, truncate short or excessively N-laden sequences, clean adapter contamination, and remove PCR duplicates. Hi-C raw data were also filtered using fastp to obtain clean data, which were then processed with HICUP (v0.8.0) to remove unmapped reads, invalid pairs (including self-circles and dangling ends), and PCR duplicates, ensuring reliable analysis results. In summary, rigorous data filtering steps were implemented for various sequencing technologies to generate high-quality datasets for subsequent analyses. The initial assembly of ultra-long sequencing data obtained from ONT was performed using nextDenovo software (v2.5.0). In summary, nextDenovo was utilized to assemble the ultra-long reads at the outset, thereby enabling the establishment of a contiguous sequence scaffold for subsequent analytical endeavors. The preliminary assembly results underwent two iterations of error correction utilizing the Racon software (v1.4.11). Subsequently, the third-generation-corrected genome underwent two further rounds of refinement through error correction, leveraging second-generation sequencing data and the Pilon software (v1.23). The corrected genome was then utilized for subsequent gap filling. In summary, a multi-round error correction approach combining third- and second-generation sequencing data was employed to enhance the accuracy of the genome assembly. Two distinct assembly approaches were utilized for HiFi data: one involved a pure HiFi assembly process using hifiasm (v0.16.1-r375), while the other incorporated a mixed assembly strategy combining HiFi and ONT ultra-long reads using hifiasm (v0.18.2-r467). Post-assembly, non-T2T scaffold versions required no further processing. However, for T2T scaffold versions, particularly those with high heterozygosity or a genome size significantly larger than expected, haplotig removal was conducted using Purge_haplotigs (v1.0.4) or Purge_dups (v1.2.5) to refine the T2T HiFi scaffold genome. In summary, tailored assembly strategies and post-processing steps were applied to ensure the quality of HiFi genome assemblies. Hi-C scaffolding was performed using ALLHIC (v0.9.8) [21] and 3D-DNA [22] pipeline. Manual refinements and error corrections were performed using medaka (v1.5.0) and deepVariant (v1.3.0) [23] to produce a chromosome-scale ginseng genome. The corrected reads were then used for the first round of gap filling on the genome, using winnowmap (v1.11) [24]. Finally, a comprehensive and rigorous evaluation of genome assembly quality was performed, employing a diverse array of metrics. The assembly quality of the chromosome-level genome was evaluated based on several criteria: continuity was assessed through contig count, N50 length, and gap count; genome completeness was gauged using BUSCO (v5.3.0) and the LTR Assembly Index (LAI); correctness was evaluated by mapping rates derived from ONT ultra-long and PacBio HiFi reads; accuracy was determined by the consensus QV obtained through K-mer spectrum comparison; and structural integrity was assessed by identifying syntenic relationships and SVs using mummer and Syri (v1.6).

### Genome annotation

Repetitive sequences were identified using two approaches: homology comparison and *de novo* prediction. For *de novo* prediction, RepeatModeler (version 2.0.4) [RepeatMasker open-4.0] was utilized to generate model sequences from the genome sequence itself. Additionally, LTR_FINDER (official release of LTR_FINDER_parallel) [25] and LTRharvest (v1.62) [26] were employed to predict LTR sequences. Subsequently, LTR_retriever (v2.9.0) [27] was used to eliminate redundancy and curate a set of non-redundant LTR sequences. The two *de novo* sequence sets were merged to form a *de novo* repetitive sequence library. Teclass (v2.1.3) was used to classify and characterize the unidentified sequences within the library. Subsequently, the RepBase library (v20 181 026) was integrated with the *de novo* library, and RepeatMasker (v4.1.5) was applied to align and annotate repetitive sequences, yielding the combined ‘Denovo + RepBase’ results. Furthermore, the RepeatProteinMask component of RepeatMasker (v4.1.5) was employed to predict TE_protein-type repetitive sequences, resulting in the ‘TE Proteins’ dataset. Ultimately, all repetitive sequence prediction outcomes were consolidated and deduplicated to produce the definitive genome-wide repetitive sequence dataset, denominated as ‘Combined TEs’.

RNA-seq reads underwent quality filtering with fastp (v0.23.2) [28] and were subsequently aligned to the genome utilizing HISAT2 (v2.2.1) [29]. The aligned results were then used by Stringtie (v2.2.1) [30] to reconstruct transcripts, which were further analyzed by TransDecoder (v5.7.0) to predict coding frames, ultimately yielding predicted coding genes. For ONT full-length analysis, NanoFilt (v2.8.0) was used for filtering, and Pychopper (v2.7.5) was employed to identify full-length sequences. These full-length sequences were mapped to the genome by using minimap2 (v2.26-r1175) [31]. TransDecoder (v5.7.0) was then used for prediction. Lastly, MAKER (v3.01.03) [32] integrated the predicted gene sets, and after multiple comparisons, decisions were made on whether to replace existing annotations from maker (v3.01.03) /EVidenceModeler (v2.1.0) [33], subsequent to the alignment, the gene set underwent additional refinement to achieve the final version. The annotational completeness of the genome was then assessed using BUSCO (v5.4.7) [34].

The protein sequences from the final determined gene set were aligned with existing databases to determine gene functions, including the Universal Protein Resource (Uniprot) (v2022-03-09) [35] and the Non-redundant Protein Database (NR) (v2022-03-09). The UniProt database documents the mapping between individual protein families and functional annotations in the Gene Ontology (GO) framework [36], as well as within the Clusters of Orthologous Groups (COG/KOG) [37]. Diamond software was utilized to annotate the biological functions performed by the protein sequences encoded by the genes. InterProScan (v5.55–88.0) [38] was employed to search against sub-databases within InterPro, resulting in the identification of conserved protein sequences, motifs, and structural domains. Furthermore, hmmscan (v3.3.2) [39] was employed for prediction of protein domains, yielding similar results regarding conserved sequences, motifs, and domains. Kofam v3.0 [40] was used to enrich KEGG (v2023-07-11) [41] orthologs and pathways. tRNAscan-SE (v2.0.12) [42] was used to annotate tRNA structures, while RNAmmer (v1.2) [43] was employed for rRNA prediction. INFERNAL (v1.1.4) [44] was used to annotate various types of non-coding RNAs, including microRNAs (miRNAs), small nuclear RNAs (snRNAs), and other ncRNAs (non-coding RNAs). Telomeres and centromeres were identified using quarTeT2 and T2Tools (accessible at https://github.com/sc-zhang/T2Tools). Subsequently, the ginseng genome was partitioned into subgenomes A and B through the application of SubPhaser.

SV and PAV detection in this project was performed by MUMmer and SyRI. MUMmer was employed for whole-genome alignment. And SyRI (Synteny and Rearrangement Identifier) was utilized to identify variations. ‘Presence’ refers to insertions greater than 30 bp, and ‘absence’ refers to deletions greater than 30 bp. PAVs are a summation of presence, absence, highly divergent regions (HDR), and not-of-type-able (NOTAL) variations.

### Phylogenetic analyses

OrthoFinder (v2.3.12) [45] was employed to detect paralogs and orthologs, and to reconstruct the species phylogeny among eleven plant species, namely *Eleutherococcus senticosus* [46], *Aralia elata* [47], *Panax stipuleanatus* [7], *Panax notoginseng, Panax quinquefolius, Panax japonicus, Panax ginseng* [48], *Vitis vinifera* [49], *Solanum lycopersicum* [50], *Oryza sativa* [51], *Angelica sinensis* [52], and *Daucus carota* [53]. Gene family clustering was performed based on the complete amino acid sequences of twelve plant species, utilizing the OrthoFinder software (v2.3.12) [45] with blastp (v2.6.0) [54] for alignment. The aforementioned species were used to identify single-copy orthologous genes. Subsequently, for each single-copy gene family, the corresponding protein sequences were subjected to multiple sequence alignment using MUSCLE (v3.8.31) [53], which is noted for its high accuracy and throughput in generating multiple sequence alignments. The alignment outcomes were refined using trimAl (v1.2rev59) [55] and subsequently merged to form a super gene. A phylogenetic tree was constructed using the maximum likelihood (ML) method implemented in RAxML (v8.2.10) [56], employing the PROTGAMMAWAG substitution model. Subsequently, this species tree was utilized as input for divergence time estimation using the mcmctree program from the PAML package [57]. Multiple fossil times from TimeTree (http://www.timetree.org/) were incorporated for time calibrations. Gene family expansion and contraction were inferred using CAFE (v3.1) [57] based on the chronogram of the aforementioned 11 plant species. Calibration points derived from TimeTree and utilized in our analysis include: the divergence between *P. stipuleanatus* and *P. notoginseng* at 45.5–52.0 Mya, *A. elata* and *P. ginseng* at 30.9–35.8 Mya, *E. senticosus* and *A. elata* at 13.3–79.8 Mya, *S. lycopersicum* and *D. carota* at 97.5–109.2 Mya, as well as *V. vinifera* and *O. sativa* at 142.1–163.5 Mya. Gene family expansions and contractions were detected using CAFE (v3.1) [58]. Subsequently, functional annotations were performed through GO and KEGG enrichment analyses.

### Determination of flavonoids

Samples were collected from the roots, stems, leaves, and three different fruit developmental stages of two *P. quinquefolius* varieties, with three biological replicates for each sample. The sample underwent freeze—drying in a freeze—dryer and was then pulverized into powder. Then, each sample was separately weighed with 50 mg powder and added with 1200 μl methanol solution. Swirl and mix well twice for 1 min each time. Subsequently, the supernatant was sucked out by centrifugation at 12 000 rpm for 3 min. Finally, the samples were passed through a 0.22-μm microporous membrane for filtration and then placed into injection vials for UPLC–MS/MS analysis.

The sample analysis was conducted using an UPLC-ESI-MS/MS system (Exion LC™AD, SCIEX). The UPLC separation was performed on an Agilent SB-C18 column (1.8 μm, 2.1 × 100 mm) with a mobile phase composed of solvent A (water containing 0.1% formic acid) and solvent B (acetonitrile containing 0.1% formic acid). The gradient elution was initiated at 95% A and 5% B, linearly adjusted to 5% A and 95% B over 9 min, and maintained at this composition for an additional 1 min. Subsequently, the composition of 95%A, 5.0%B was adjusted and held for 2.9 min within 1.1 min. The flow rate was set at 0.35 ml per minute; Column oven set at 40°C; 2 μl injection volume. The eluent is alternately directed to an ESI-Triple Quadrupole Linear Ion Trap-MS for detection.

### RNA extraction, cDNA library construction, and transcriptome assay were performed

Total RNA was isolated from each sample using the TRIzol kit (Invitrogen, CA, USA). The concentration and quality of the purified RNA were assessed using a Qubit 4.0 fluorometer /MD enzyme labeler and a Qsep400 bioanalyzer. Reverse transcription was carried out using the purified mRNA fragments as templates. Then the purified double-stranded cDNA was subjected to end repair, followed by the addition of an A-tail and the ligation of sequencing adapters. Subsequently, the fragment size was selected using DNA purification magnetic beads, and finally PCR amplification was conducted to generate the final cDNA library. After successful validation of the library quality, distinct libraries were combined through pooling based on the target sequencing depth, and sequencing was subsequently performed on the Illumina platform.

### WGCNA analysis

The input fpkm expression files were filtered, varFilter function of R package genefilter was used to remove low-expression and stably expressed genes in all samples, and the soft threshold was set to 0.8. A hierarchical clustering tree was assembled based on the correlation of gene expression levels, and modules were divided.

### Functional annotation of genes and identification of differentially expressed genes

The expression levels of transcripts or genes were quantified using the fragments per kilobase of transcript per million mapped reads (FPKM) metric. Differential expression analysis between the two groups was conducted using DESeq2, and the resulting *P*-values were adjusted for multiple testing using the Benjamini–Hochberg correction method. After correction |log2FC| ≥ 1, and a false discovery rate < 0.05 was used as the threshold for significant differential expression [59]. Subsequently, GO and KEGG functional enrichment analysis was performed using the ClusterProfiler R software package by comparing the differential genes to the reference genome, and the significantly enriched terms were identified based on the hypergeometric test (*q*-value <0.05).

### Prediction of TFs and construction of co-expression networks

To identify TFs, all annotated genes were compared against the Plant Transcription Factor Database (PlantTFDB v.4.0), utilizing *A. thaliana* protein sequences as reference standards. Gene co-expression networks were established based on the identified DEGs and TF using WGCNA package. Additionally, the interaction relationships among these genes were analyzed through the STRING protein interaction database (http://string-db.org) to elucidate differential gene protein interaction networks. This approach facilitated the construction of a co-expression regulatory network associated with anthocyanin biosynthesis. Data were visualized using Cytoscapev.3.5.1 [60].

## Supplementary Material

Web_Material_uhaf198

## Data Availability

The raw transcriptome sequence data for *P. quinquefolius* have been deposited in the National Center for Biotechnology Information (NCBI), assigned with the BioProject number PRJNA1226493. Additionally, the genome assembly data for *P. quinquefolius* have been archived in the China National Center for Bioinformation, under the project number PRJCA036445. All pertinent data related to this study are included in the supplementary materials that accompany this paper.
