# Supplementary material for: Telomere-to-telomere genome assembly of yellow-fruited allotetraploid American ginseng (Panax quinquefolius L.) provides insights into flavonoid biosynthesis
Source: Hortic Res. 2025 Jul 29;12(10):uhaf198. doi: 10.1093/hr/uhaf198 (PMC12549079; doi:10.1093/hr/uhaf198)
Supplement: Web_Material_uhaf198 [file web_material_uhaf198.zip › HR-2025-116_Figure S.docx]

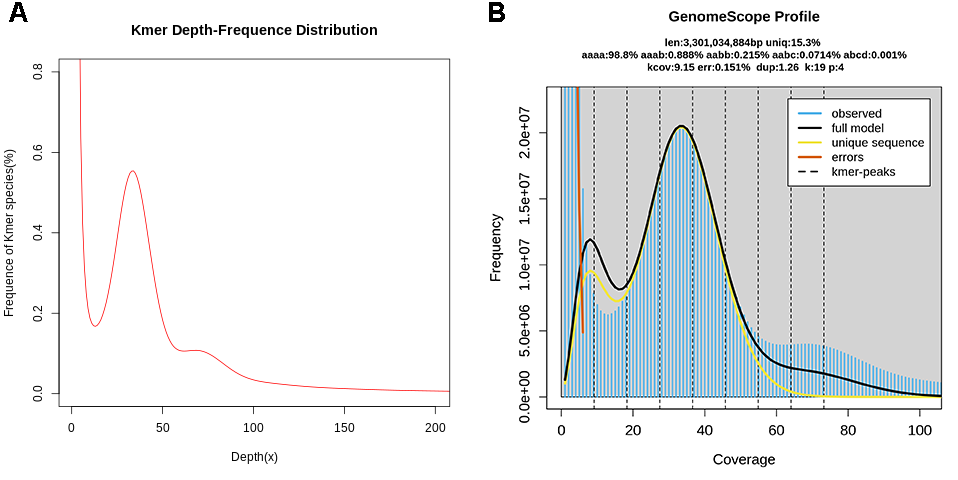
Figure S1

**Figure S1. Genome Survey result of *P. quinquefolius.* A** Estimation of *P. quinquefolius* genome size by *K*-mer analysis. *x*-axis shows *K*-mer depth and *y*-axis shows *K*-mer frequency. **B** GenomeScope Analysis of *P. quinquefolius* : estimating genomiccharacteristics from sequencing data.

Figure S2


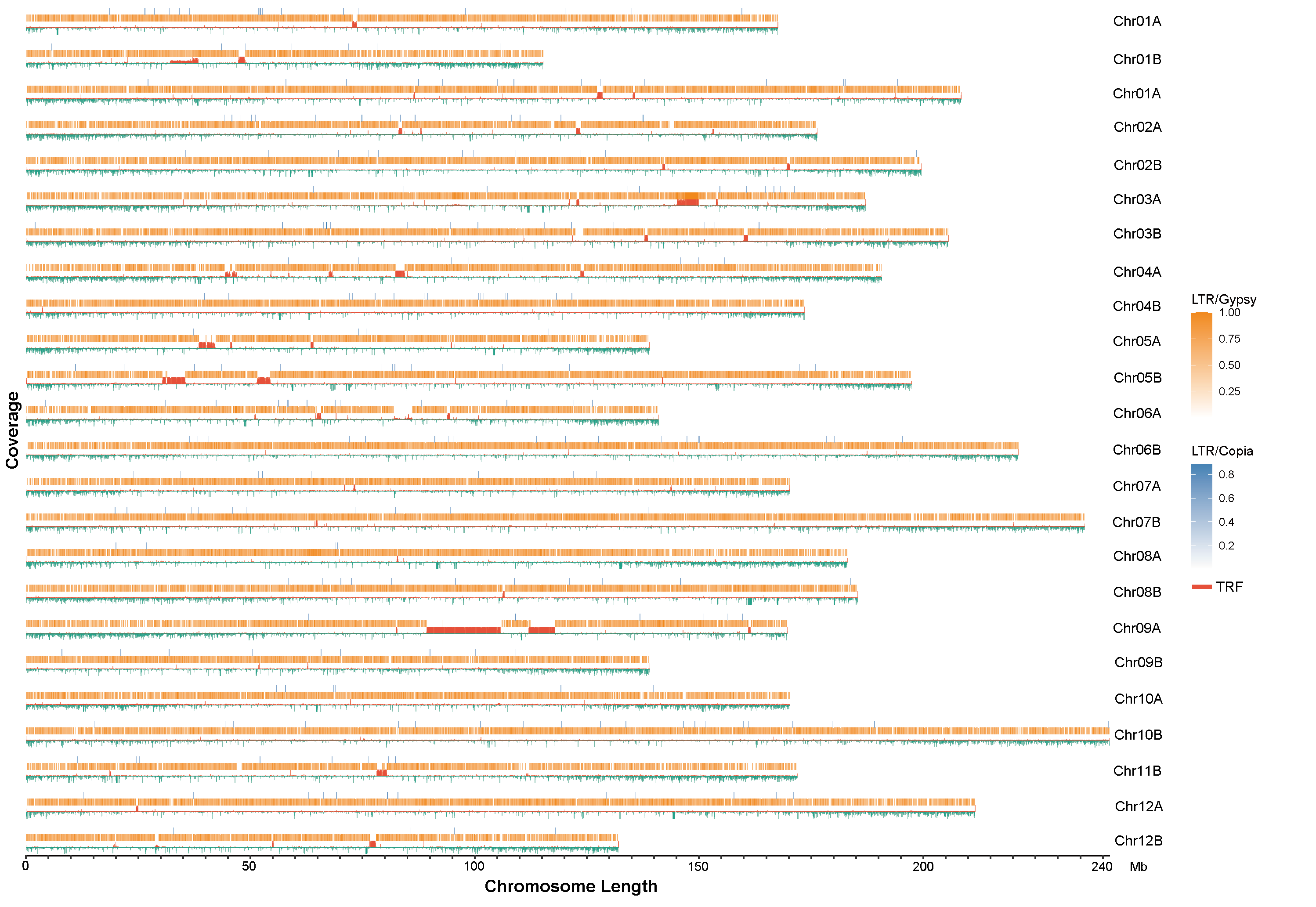
**Figure S2. The predicted chromosomal model of *P. quinquefolius*.** The distribution of predicted centromere and LTR density regions. Orange represents the coverage of LTR/Gypsy, blue represents the coverage of LTR/Copia, and red represents TRF.

Figure S3


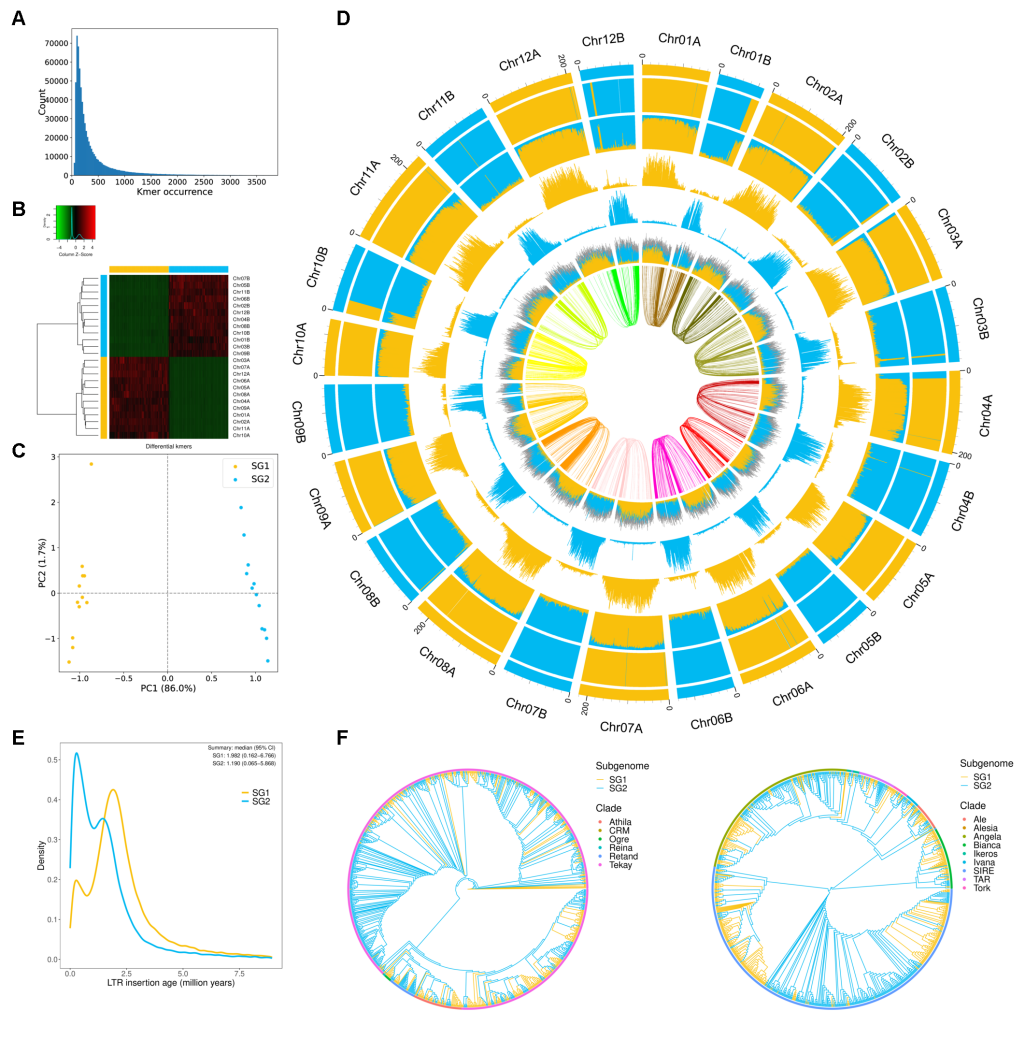


**Figure S3. Subgenome (SG) phasing and characterization of the allotetraploid *P.quinquefolius* genome. A** The number of differential 19-mers among homoeologous chromosomes. **B** Unsupervised hierarchical clustering is employed, with the horizontal color bar atop the axis designating the specific subgenome to which each k-mer is associated, and the vertical color bar to the left of the axis indicating the subgenome assignment for each chromosome. The heatmap visually represents the Z-scale relative

abundance of k-mers, where a higher Z-score implies a greater relative abundance of the corresponding k-mer. **C** PCA of variable19-mers confirms genome segmentation into SG1 and SG2, based on patterns in k-mers and homoeologous chromosomes. **D** Chromosomal Characteristics. Proceeding from the outermost circle towards the innermost (1–7): (1) Assignment of subgenomes based on the application of the k-means algorithm; (2) Significant enrichment of subgenome-specific k-mers, indicated by the use of colors corresponding to those of the respective subgenomes. The presence of the same color suggests a notable concentration of such subgenome-specific k-mers, whereas white areas signify no significant enrichment; (3) A normalized proportion (relative) of subgenome-specific k-mers is presented; (4-5) The count (absolute) of each set of subgenome-specific kmers is detailed; (6) The density of Long Terminal Repeat Retrotransposons (LTR-RTs) is displayed. If the color aligns with that of the subgenome, it denotes a significant enrichment of LTR-RTs corresponding to those subgenome-specific k-mers. Gray signifies the presence of nonspecific LTR-RTs; (7) Homoeologous blocks. **E** Insertion timing of subgenome-specific LTR-RTs, with a 95% Confidence Interval (CI) in top right for estimating insertion time span. **F** Phylogenetic tree of 1000 random Gypsy LTR-RTs and different transposon families from subgenome-specific sets, colored by subgenome (branches) and clade (terminal nodes) by TEsorter. SG1 represents Subgenome A, SG2 represents Subgenome B.


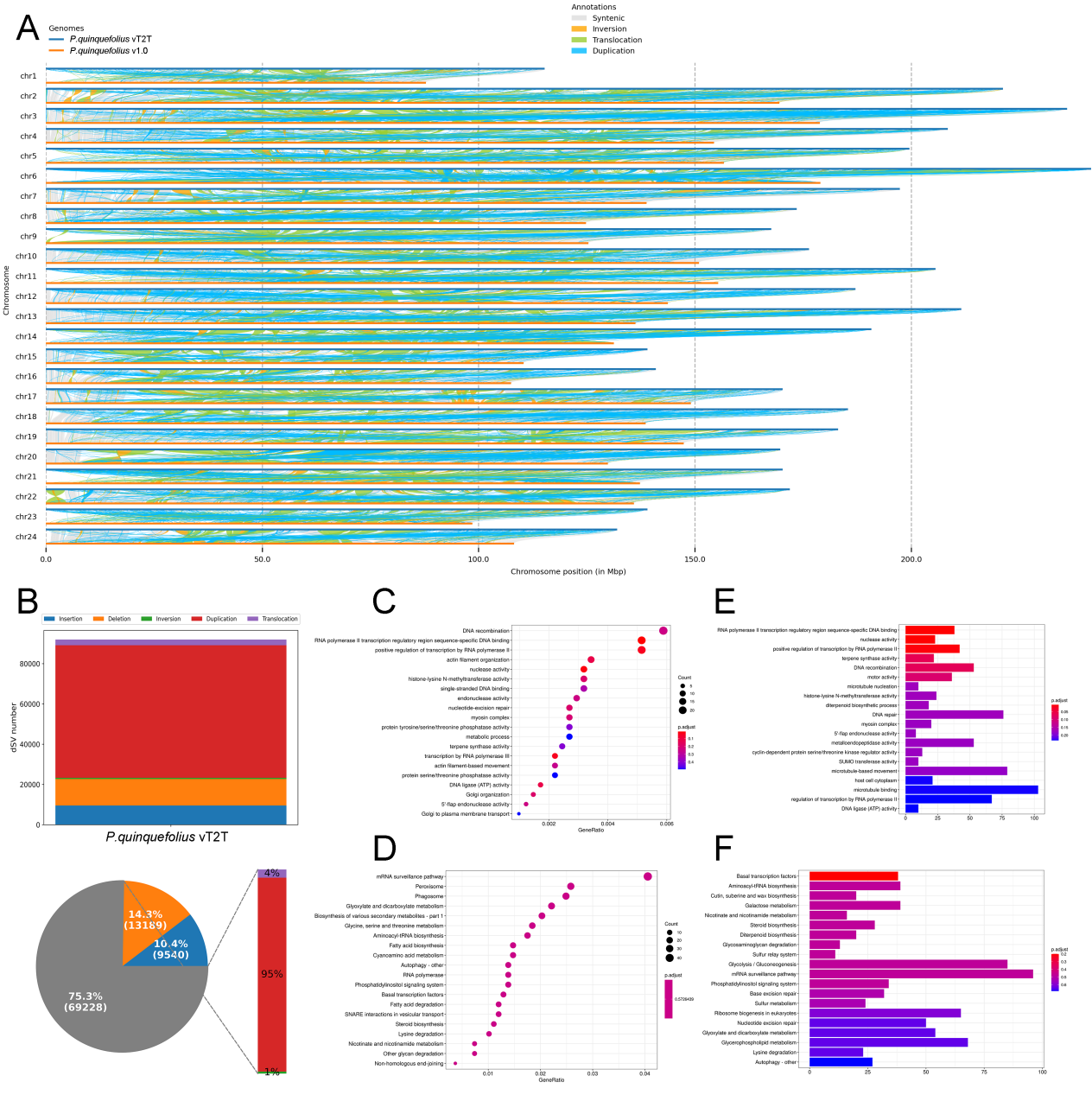
Figure S4

**Figure S4. Structural comparison between *P.quinquefolius* vT2T and *P. quinquefolius* v1.0*.* A** Synteny and rearrangement identification between two genome versions. Gray represents collinearity, orange represents inversion, green represents translocation, and blue represents duplication. **B** The classification and number of structural variations. Blue represents insertion, orange represents deletion, green represents inversion, red represents duplication, and purple represents translocation. **C** GO enrichment of structural variations. **D** KEGG enrichment of structural variations. **E** GO enrichment of presnece/absence variation. **F** KEGG enrichment of presnece/absence variation. The *x*-axis represents the gene ration, while the *y*-axis indicates the gene ontology (C) or KEGG pathway (D). The color denotes the *p.adjust* value.


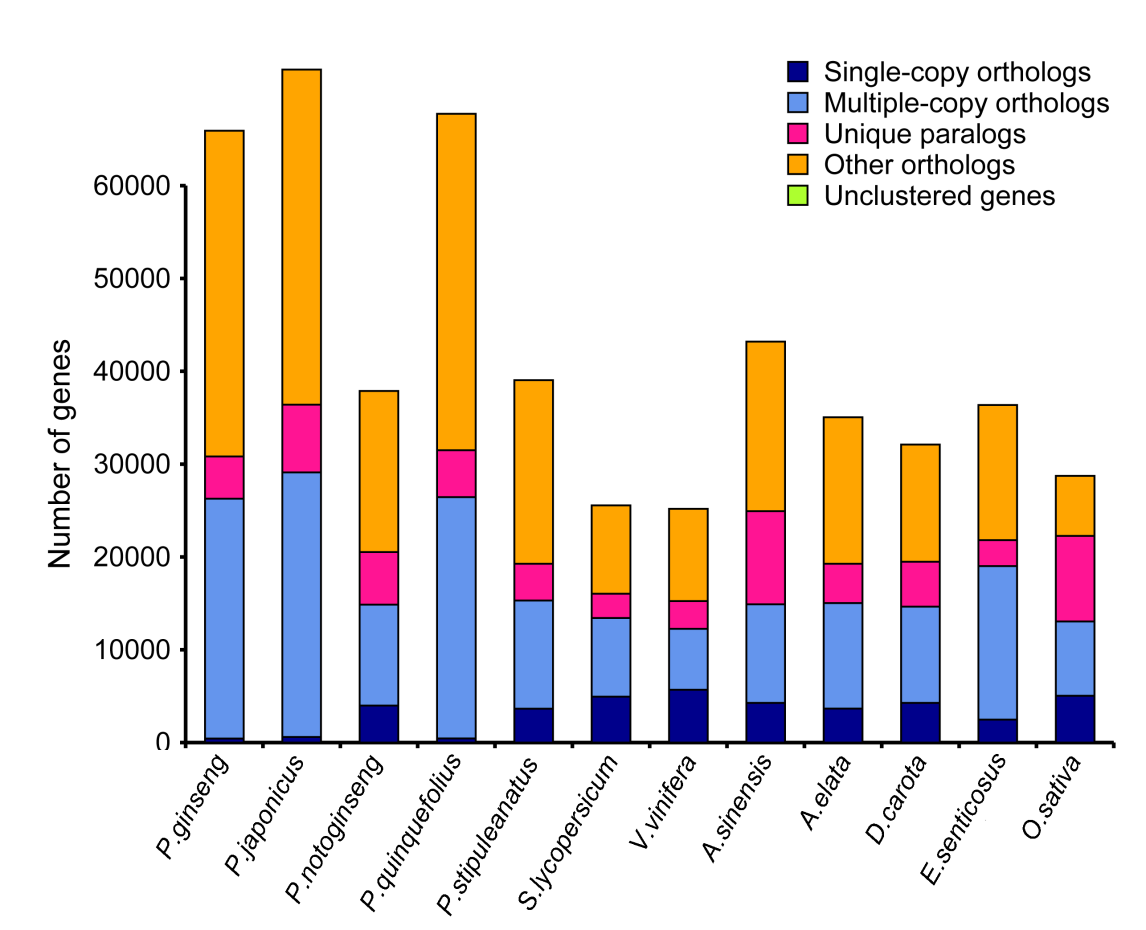
Figure S5

**Figure S5. The number of homologous Genes in different species.**

Figure S6


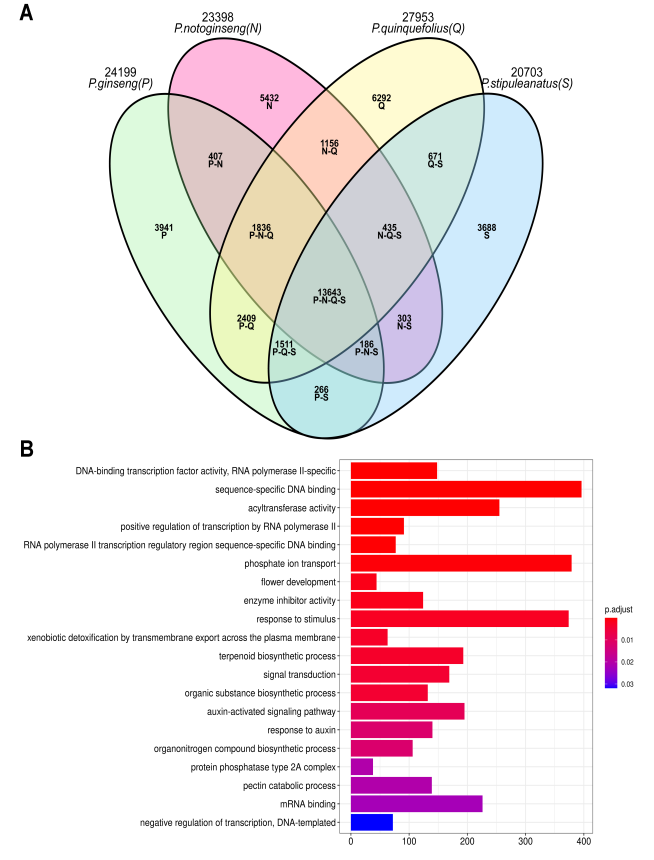
**Figure S6. Venn diagram and GO enrichment of gene families across four species of the Araliaceae family.A** The four species of the Araliaceae family are *P.ginseng (P), P.notoginseng (N)*, *P.quinquefolius (Q)*, and *P.stipuleanatus (S)*, with the numbers indicating the count of gene families. The letters in parentheses are abbreviations of the Latin names for these species. The concatenated strings of abbreviations represent the gene families shared among these species. A single abbreviation letter indicates the number of unique gene families in that species among the four. **B** Barplot of shared gene across four species of the *Araliaceae* family with GO enrichment. The *x*-axis represents the number of genes enriched, while the *y*-axis indicates the gene ontology. The color denotes the *p.adjust* value.


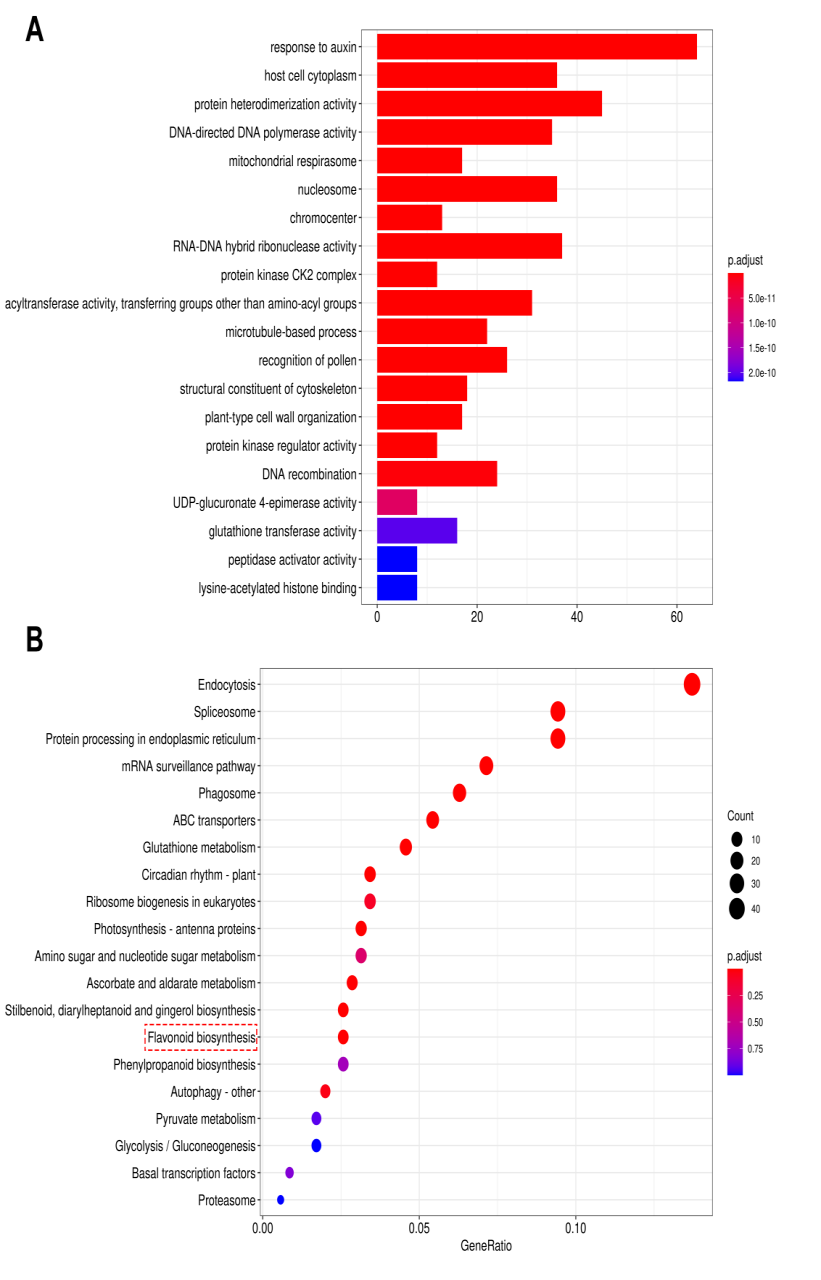
Figure S7

**Figure S7. GO and KEGG enrichment of expansion genes in *P. quinquefolius.* A** Barplot of expansion genes with GO enrichment. The *x*-axis represents the number of genes enriched, while the *y*-axis indicates the gene ontology. **B** KEGG enrichment of expansion genes. The *x*-axis represents the number of genes enriched, while the *y*-axis indicates the KEGG pathway.The color denotes the *p.adjust* value.

Figure S8


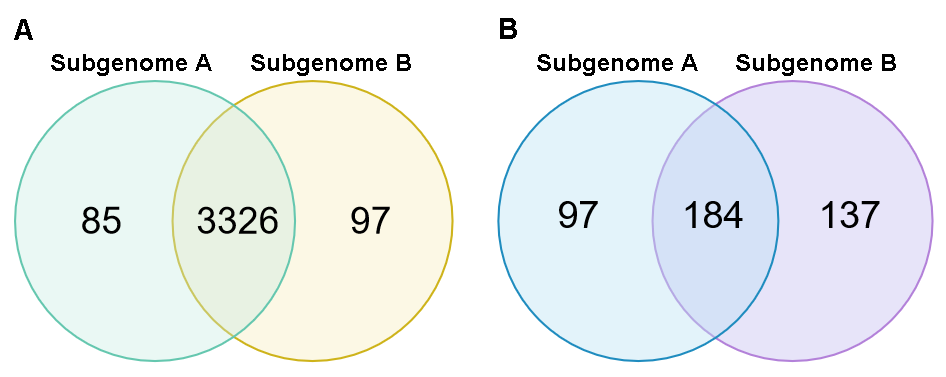


**Figure S8. Venn diagram of gene family expansion and contraction of subgenome A and subgenome B in *P. quinquefolius.* A** Venn diagram of gene family expansion of subgenome A and subgenome B in *P. quinquefolius.* **B** Venn diagram of gene family contraction of subgenome A and subgenome B in *P.quinquefolius.*


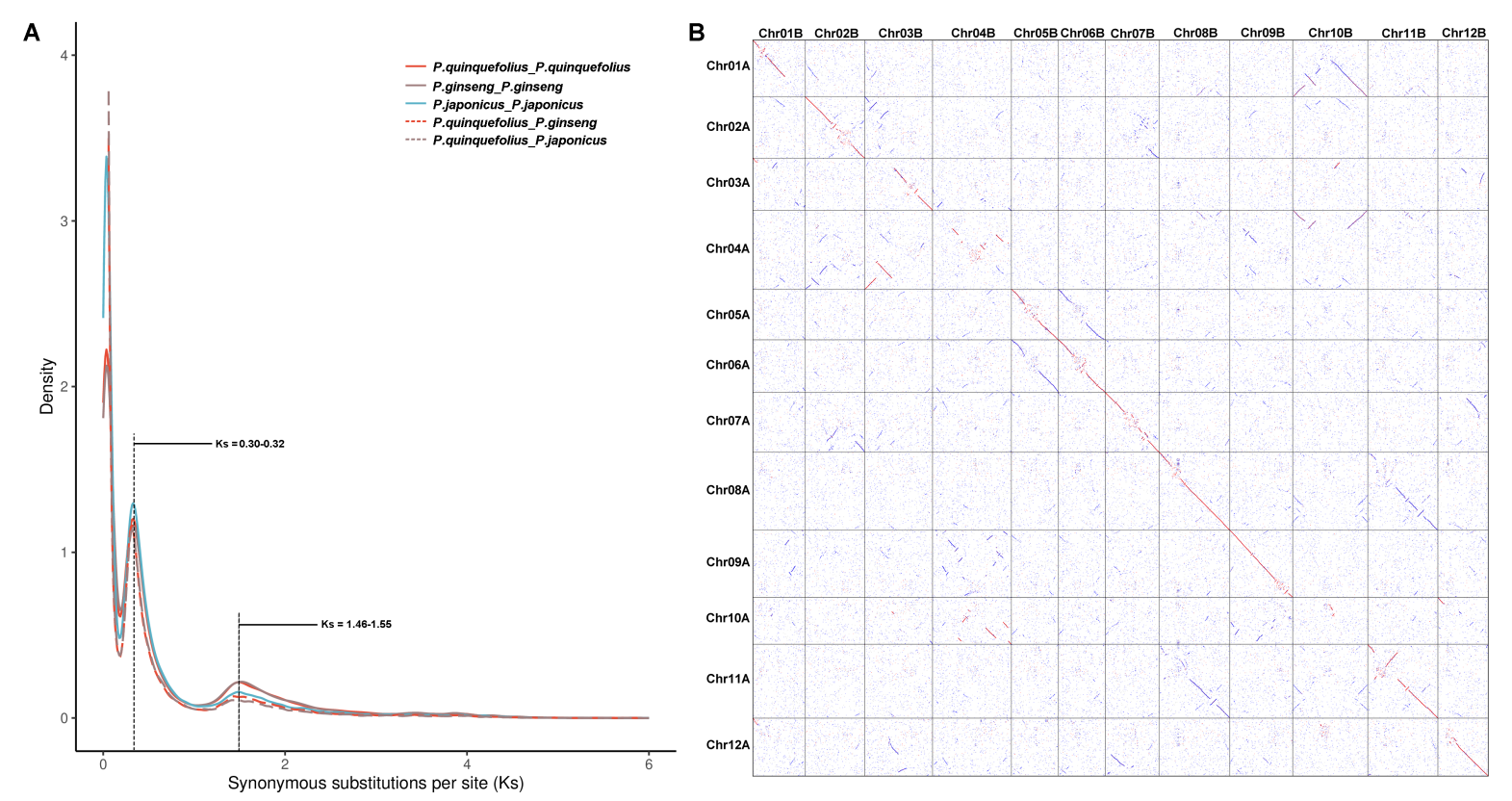
Figure S9

**Figure S9. Comparative genomic analyses in *P. quinquefolius* and other species*.* A** Synonymous substitutions per synonymous site (ks) plot of WGD events detection of *P. quinquefolius* and other species*.* **B** Dot plot visualization of collinearity between 24 chromosomes.


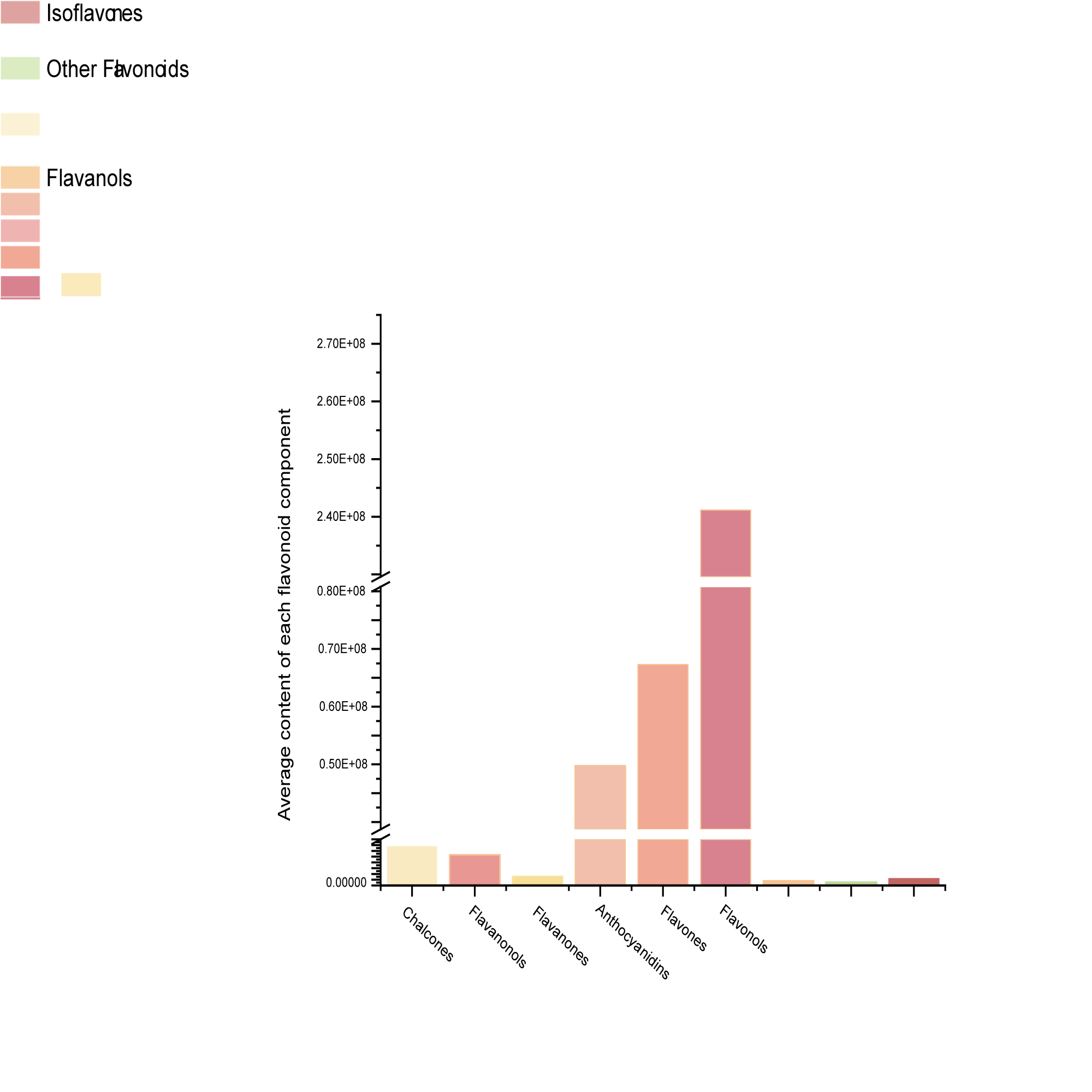
Figure S10

**Figure S10. Content of various flavonoid metabolites in *P. quinquefolius*.**


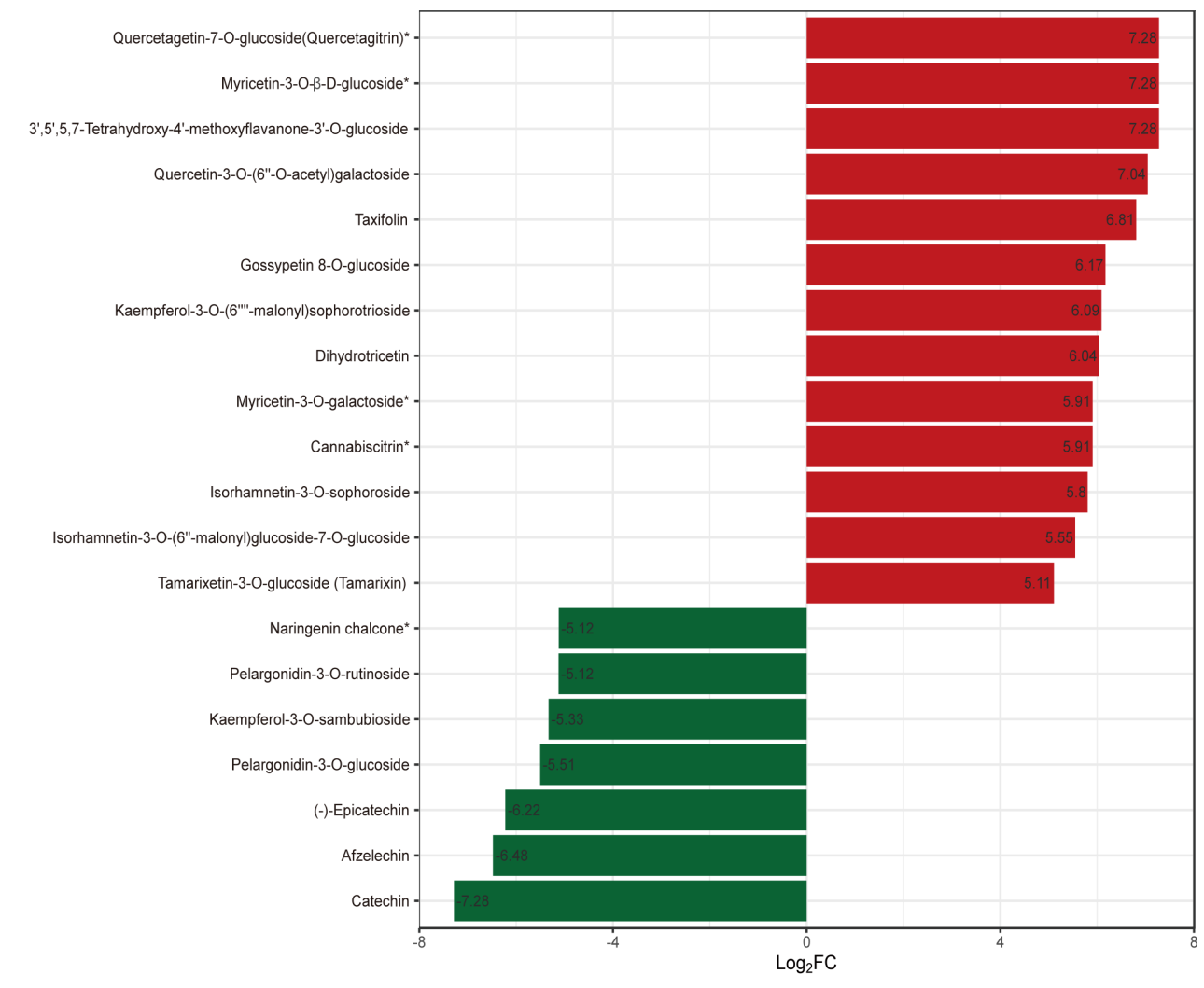
Figure S11

**Figure S11. the top 20 different accumulated flavonoid in fruits of two *P. quinquefolius* varieties**

Figure S12


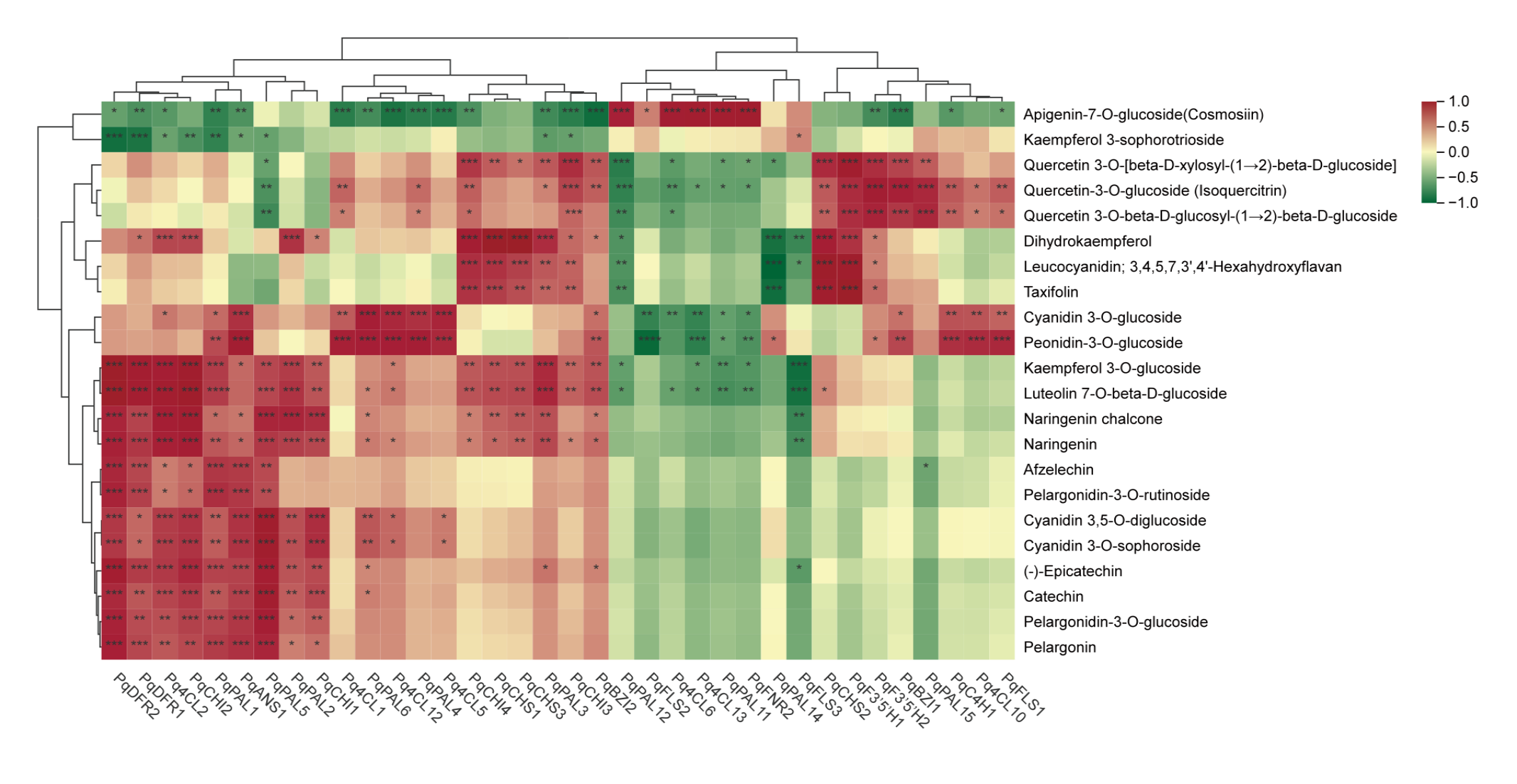
**Figure S12. Correlation analysis of flavonoid metabolites and biosynthetic genes during the fruit development of *P. quinquefolius.***

***
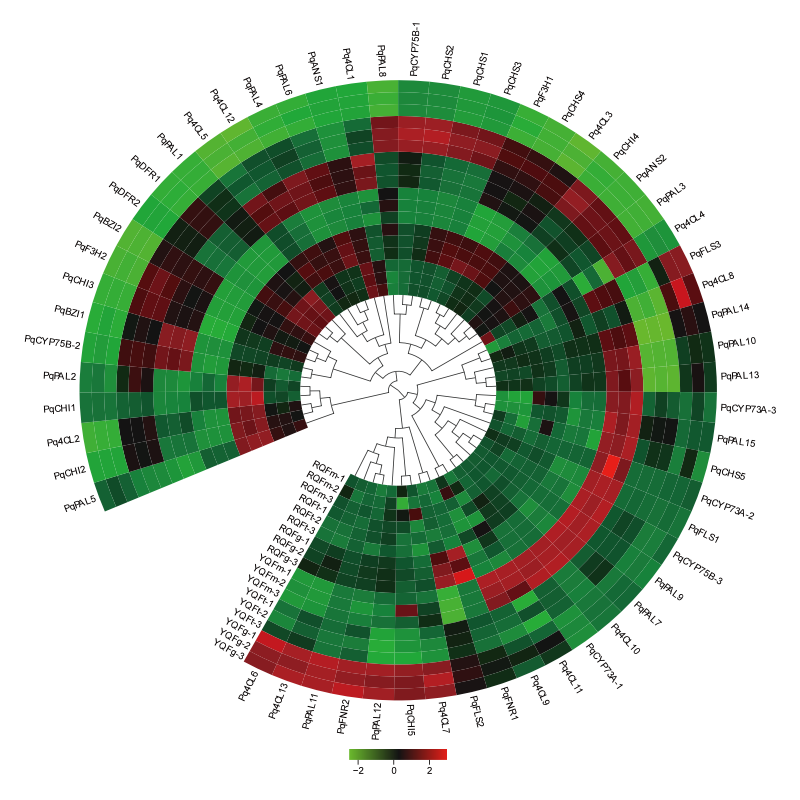
***Figure S13

**Figure S13. Clustering heatmap of the relative expression levels of flavonoid genes associated with 'Jiyue No1'(JY) and 'Zhongnongyangshen No2'(ZN) during fruit development.**

***
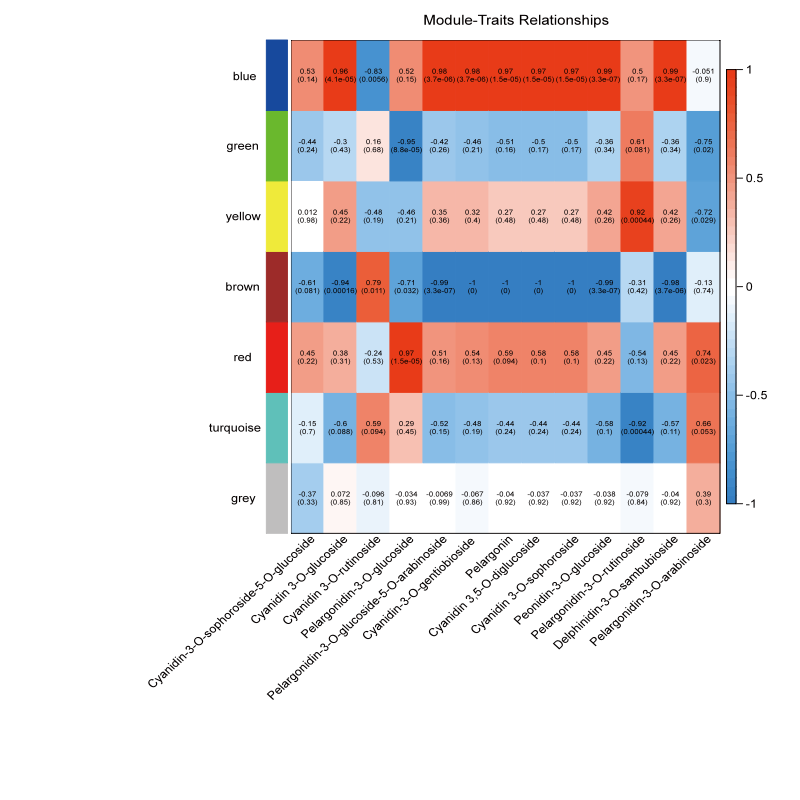
***Figure S14

**Figure S14. Weighted gene co-expression network analysis of gene expression and anthocyanin composition in fruits of ZN at different developmental stages.**

Figure S15

**Figure S15.KEGG enrichment analysis. A KEGG enrichment analysis of the green module in the weighted gene co-expression network analysis of JY. A KEGG enrichment analysis of the blue module in the weighted gene co-expression network analysis of ZN.**


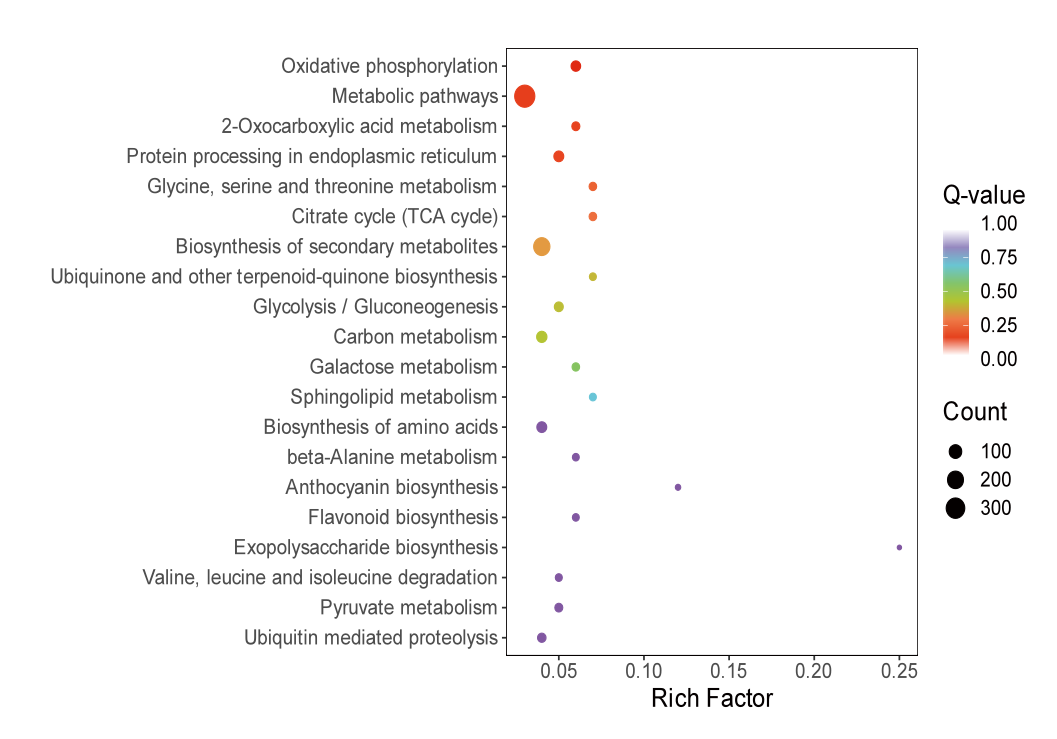

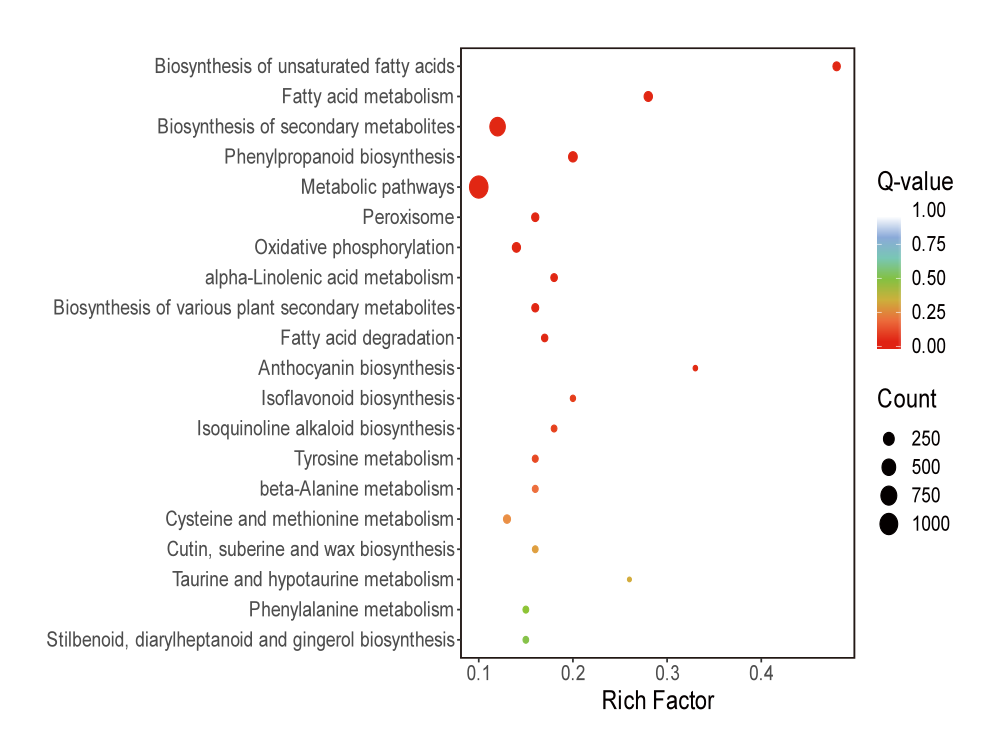


A B

Figure S16

***
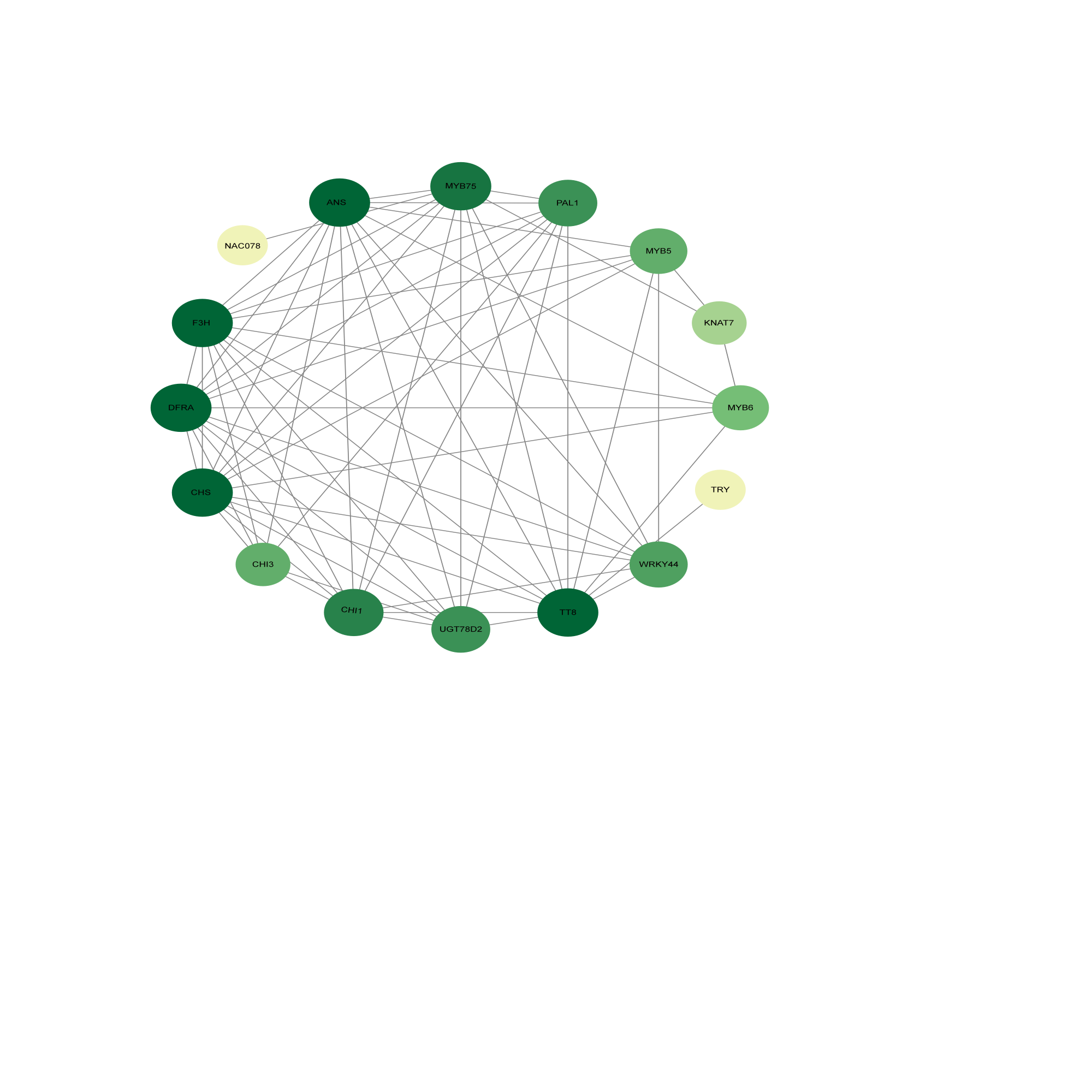
*Figure s16. construction of the regulatory network for anthocyanin biosynthesis**

Figure S17

***
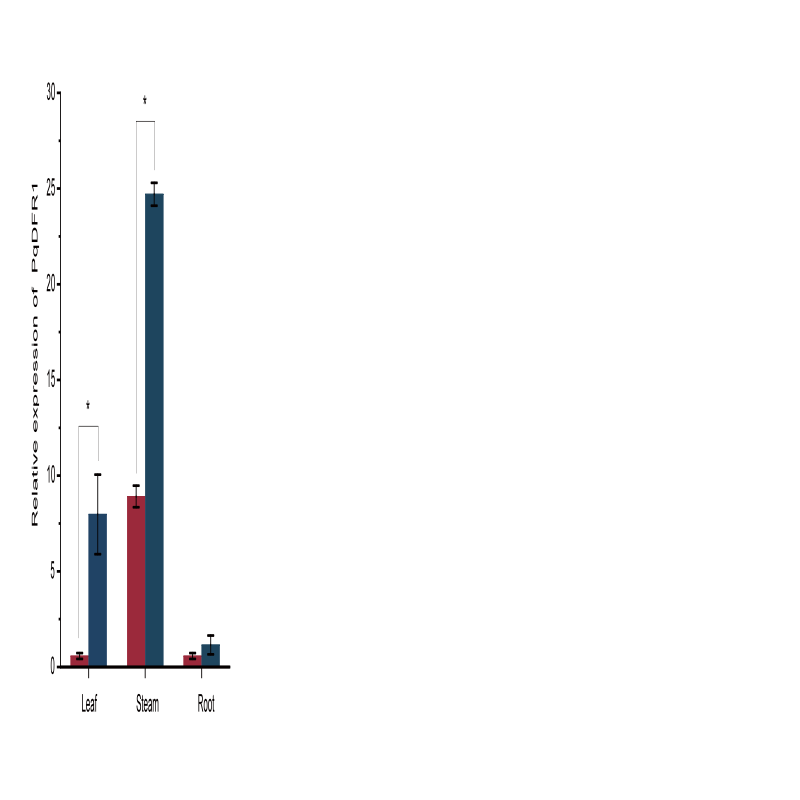
*** **Figure S17. qRT-PCR analysis of the expression of three anthocyanin-related candidate genes in the roots, stems and leaves of two *P. quinquefolius* varieties.'Jiyue No1'(JY) and 'Zhongnongyangshen No2'(ZN).**
